# Rare Earth Elements and Technology-Related Trace Metals in Paediatric Scalp Hair: A 2001 Urban Baseline from Spain

**DOI:** 10.3390/jox16010038

**Published:** 2026-02-23

**Authors:** Antonio Peña-Fernández, Manuel Higueras, Roberto Valiente Borox, M. Carmen Lobo-Bedmar

**Affiliations:** 1 Department of Surgery, Medical and Social Sciences, Faculty of Medicine and Health Sciences, University of Alcalá, Ctra. Madrid-Barcelona, Km. 33.600, Alcalá de Henares, 28871 Madrid, Spain; 2 Scientific Computation Research Institute (SCRIUR), Universidad de La Rioja, 26006 Logroño, Spain; 3 Department of Geology, Geography and Environment, Faculty of Philosophy and Letters, University of Alcalá, Calle Colegios 2, Alcalá de Henares, 28801 Madrid, Spain; 4 Madrid Institute for Rural, Agricultural and Food Research and Development (IMIDRA), Departamento de Investigación Agroambiental, Finca el Encín, Crta. Madrid-Barcelona, Km. 38.2, Alcalá de Henares, 28800 Madrid, Spain

**Keywords:** rare earth elements, emerging trace metals, hair biomonitoring, children, adolescents, reference values, Spain

## Abstract

Rare earth elements (REEs) and technology-related trace elements are increasingly used in modern products and processes, but biomonitoring data in healthy children and adolescents remain scarce; scalp hair provides a practical, integrative matrix for assessing multi-element patterns over time. Scalp hair collected in April–May 2001 from children (6–9 years; n = 120) and adolescents (13–16 years; n = 97) living in Alcalá de Henares (Spain) was retrieved from archival storage and analysed in 2025 using a single QA/QC-controlled ICP–MS workflow. Seven REEs (Ce, La, Pr, Nd, Gd, Er, and Y) and nine technology-related trace elements (Bi, Sb, Th, U, Pd, Pt, Rh, Ir, and Rb) were quantified after rigorous decontamination; left-censored data were treated using Kaplan–Meier, regression on order statistics, and maximum-likelihood approaches, and population reference values were derived as percentile-based upper limits (P95, 95% CI). In children, REEs were frequently detected and showed strong within-suite covariation, with medians in the low ng g^−1^ range (e.g., Ce ≈ 0.011 µg g^−1^; La ≈ 0.007 µg g^−1^), whereas in adolescents, most REEs were near reporting limits. Sb and U were ubiquitous in both age groups, while platinum-group elements were largely undetected. Shale-normalised REE patterns were subparallel across normalisers, La/Ce anomalies were centred below unity, and weak soil–hair correlations suggested multiple microenvironmental exposure pathways. These data provide a robust pre-diffusion baseline for REE metals in European youth, offering a benchmark for future urban exposome assessments.

## 1. Introduction

Rare earth elements (REEs), which comprise the fifteen elements of the lanthanide series together with scandium and yttrium (Y), owing to their similar physicochemical properties, as well as a suite of emerging trace metals, are increasingly integral to modern technologies, transport, and consumer products, raising concerns about inadvertent human exposure and potential health effects. These elements are pivotal for the transition to a low-carbon economy, being used in a wide range of sectors including energy, electronics, defence, and the environment [1,2,3]. Beyond their technological importance, REEs have now been established as emerging environmental contaminants because mining, smelting, and recycling activities release them into the air, water, soil, and sediments, enabling multiple routes of human exposure [4,5].

Humans can absorb REEs through inhalation, ingestion, and dermal contact, after which they are distributed systemically and accumulate in tissues such as bone, liver, kidney, lung, and brain, and in keratinised matrices including hair and nails [5]. For non-occupationally exposed populations, dietary intake represents the dominant pathway, as measurable levels of REEs have been reported in seafood, cereals, mushrooms, vegetables, and even ready-to-eat baby purées sold in European markets [6,7]. Although current dietary intakes described by these authors are below toxicological concern—typically well under the tolerable daily intake proposed by the Chinese Scientific Committee (51.5 µg kg^−1^ body weight day^−1^; [8])—the continued global expansion of REE applications in green and digital technologies necessitates periodic re-evaluation of potential cumulative exposures. Experimental and occupational studies indicate that chronic or high-level exposure can cause oxidative stress, lipid peroxidation, pulmonary and renal fibrosis, male infertility, neurobehavioural alterations, and systemic organ dysfunction [5,9], reinforcing concerns about possible adverse human health effects.

Although REEs such as cerium (Ce), lanthanum (La), praseodymium (Pr), neodymium (Nd), gadolinium (Gd), and erbium (Er) are widely used in electronics, magnets, and catalysts, human biomonitoring datasets remain scarce, particularly for children and adolescents. Gaman et al. [10] provided one of the few biomonitoring assessments of REEs in European children, underscoring how rare such datasets currently are. To our knowledge, virtually no paediatric hair datasets for REEs exist in Europe; most available investigations have been conducted in highly exposed populations residing near mining or industrial areas in China, where hair and urine have been used as biomarkers, whereas European studies have largely focused on serum or blood measurements in small paediatric cohorts, including cord blood from Spanish newborns [10,11]. Reference values for REEs in children, therefore, remain unestablished. Reported concentrations vary widely depending on geography and proximity to industrial sources. Moreover, most available biomonitoring studies have analysed REEs in blood, serum, or urine, while investigations using hair are still scarce, despite its potential to provide complementary exposure information. Thus, studies conducted in mining regions have shown elevated REE levels in children’s hair and suggested age-, sex-, or area-related differences, yet paediatric reference values are still not well established [2,12,13].

Hair can serve as a useful matrix for assessing medium- to long-term exposure to metals and metalloids, since it reflects the incorporation of elements into keratin during growth and allows non-invasive collection. When appropriately decontaminated, hair provides a practical matrix for assessing longer-term exposure patterns, recognising that contributions can be both endogenous and exogenous and may differ by element. Recent controlled experiments demonstrated that, even under conditions of high ambient particulate matter, external contamination contributes minimally to most metal(loid) concentrations when rigorous washing protocols are applied [14]. For REEs, however, systematic validation of hair as a biomonitor remains limited, and further comparative analyses with blood, urine, and nails are warranted [5]. Hair analysis offers several advantages for population studies: it reflects longer exposure windows than urine or serum, facilitates storage and transport, and is particularly suitable for children and adolescents, in whom invasive sampling is often restricted [15,16]. Moreover, recent occupational research has confirmed that REEs accumulate in keratinised tissues such as hair and nails, supporting their utility as markers of both environmental and occupational exposure [5]. Although some studies have analysed REEs in paired matrices (e.g., hair and blood), a general and reproducible hair–blood relationship is not established, particularly at background exposure levels, and matrix concordance is element- and context-dependent. Consequently, hair biomonitoring represents a practical and informative approach for evaluating cumulative and historical exposure to REEs in vulnerable populations.

Beyond their technological importance, a growing body of evidence points to the potential adverse health impacts of REEs. Experimental studies consistently demonstrate that these metals can induce oxidative stress, lipid peroxidation, mitochondrial dysfunction, apoptosis, genotoxicity, and inflammatory responses, resulting in structural and functional damage in vital organs such as the liver, kidney, brain, and lungs [3,4,17]. Systematic reviews further indicate that REE exposure may disrupt endocrine and immune homeostasis, impair neurodevelopment and reproductive function, and alter gene expression through epigenetic and transcriptional mechanisms [17]. These effects appear to depend strongly on each element’s physicochemical characteristics—including valence state, ionic radius, particle size, solubility, and speciation—reflecting distinct toxicokinetic and toxicodynamic profiles across the lanthanide series [18,19,20]. Accordingly, light REEs (La, Ce, Pr, Nd) are generally more readily assimilated than heavy REEs (Gd, Er) in aquatic organisms [21], while speciation and complexation with dissolved organic matter strongly influence bioavailability and toxicity [19].

Particularly concerning are effects occurring during critical windows of susceptibility, such as pregnancy and early childhood, when the developing placenta and brain are highly vulnerable to xenobiotic interference. Recent prospective data from the Wuhan birth cohort demonstrated that higher prenatal urinary concentrations of several REEs also quantified in the present study—specifically Ce, La, Pr, Nd, Gd, and Er—were significantly associated with lower mental and psychomotor development scores in children at 24 months of age. The study identified the first and third trimesters as sensitive periods of susceptibility, with La and Ce contributing most strongly to joint mixture effects on psychomotor performance [22]. Collectively, these findings indicate that REEs, once regarded as biologically inert, may have subtle yet clinically relevant impacts on pregnancy outcomes, neurodevelopment, and systemic health [4,17].

Although broader epidemiological evidence remains limited, recent reviews emphasise that REEs now enter the human body through multiple interacting pathways, including environmental contamination of air, soil, and water; dietary intake; medical and imaging applications; and emerging emission sources such as e-waste recycling, diesel exhaust containing nano-ceria, and tobacco or e-cigarette smoke, highlighting their growing ubiquity within the human exposome [7,17,18]. Despite the expanding toxicological evidence base, there remains a critical lack of epidemiological and biomonitoring data for non-occupationally exposed populations, particularly for vulnerable groups such as children and adolescents living in urban or industrial environments, which underscores the urgent need for locally derived reference values and risk assessment frameworks.

Alongside REEs, children are increasingly exposed to a suite of emerging trace elements that originate from modern technologies and urban activities. Platinum-group elements such as palladium (Pd), platinum (Pt), rhodium (Rh), and iridium (Ir) are primarily released from automotive catalytic converters and urban traffic [23], while antimony (Sb) and bismuth (Bi) are used in flame retardants, alloys, and electronic components [24]. Uranium (U) and thorium (Th) occur naturally in soils and rocks but can be mobilised by industrial processes and waste management activities [25]. These elements are now being detected in air, dust, soil, and food and are recognised as emerging environmental contaminants because of their expanding industrial and medical applications and their potential for bioaccumulation and toxicity [3,18]. Experimental and epidemiological evidence indicates that exposure to Pd, Pt, and Rh can induce oxidative stress, inflammatory and immunomodulatory responses, renal and cardiovascular alterations, and reproductive effects, largely mediated by oxidative and apoptotic pathways [26]. Likewise, Th and U can accumulate in bone and soft tissues, causing nephrotoxicity, genotoxicity, and radiological damage in chronically exposed populations [25,27]. Human hair has been shown to be a suitable matrix for screening exposure to several of these elements, including Pd, Pt, and Rh in populations exposed to vehicular and industrial emissions [28] and Th and U in communities affected by geogenic or radiological sources [25,27]. For other trace metals such as Ir, Rb, Sb, and Bi, human biomonitoring data remain scarce. Recent reviews emphasise the importance of well-designed paediatric biomonitoring studies that report detection frequencies, distributional summaries, and appropriate treatment of censored data, and that clearly distinguish population reference values from health-based guidance thresholds [3,18,29]. They also highlight the urgent need for harmonised analytical methods, quality-assurance frameworks, and comparable datasets to strengthen exposure assessment and trend evaluation in children and adolescents.

From an analytical perspective, REEs and technology-related trace elements occur at ultra-trace levels in biological matrices and are therefore commonly quantified using inductively coupled plasma–mass spectrometry (ICP-MS) following matrix-specific pre-treatment, including rigorous decontamination of keratinised samples and acid digestion under strict quality assurance/quality control (QA/QC). Reported concentrations and detection frequencies can vary substantially across studies because they depend not only on exposure context but also on sample preparation, instrumental sensitivity, and the handling of left-censored data near reporting limits. Consequently, published background “concentration standards” or harmonised reference ranges for many REEs in healthy paediatric populations—particularly in scalp hair—remain limited across matrices (hair, blood/serum, urine, nails), motivating the percentile-based reference limits derived here.

In this study, we report hair concentrations of seven REEs (Ce, La, Pr, Nd, Gd, Er, and Y) and nine emerging trace metals (Bi, Sb, Th, U, Pd, Pt, Rh, Ir, and Rb) in healthy children aged 6–9 years and adolescents aged 13–16 years residing in Alcalá de Henares, Spain. Because samples were collected in 2001, this dataset provides a unique urban baseline predating the large-scale expansion of REE-enabled consumer technologies. The specific objectives of this work were to: (a) describe detection frequencies and distributional statistics; (b) assess age- and sex-related differences; (c) examine spatial patterns across different characteristic residential zones; and (d) propose local reference intervals for the detected elements.

## 2. Materials and Methods

### 2.1. Study Area and Population

The World Heritage City of Alcalá de Henares (40°29′ N, 3°22′ W) is a medium-sized municipality (approx. 200,000 inhabitants) located 30 km east of Madrid (central Spain) [30]. The area has a continental Mediterranean climate and mixed land use comprising dense urban districts, traffic corridors, peri-urban residential zones, and light industrial estates. Historical industrial activity, vehicular emissions, and proximity to major transport routes have led to heterogeneous metal deposition patterns documented in soils and atmospheric particulates [31].

### 2.2. Hair Sampling

Healthy schoolchildren aged 6–9 years (n = 120; 70 girls) and adolescents aged 13–16 years (n = 97; 68 girls) born and residing in Alcalá de Henares (Madrid Region, Spain) were recruited for this investigation. Participants were selected according to strict inclusion criteria designed to minimise confounding factors known to influence the elemental composition of hair [32]. All volunteers were of Caucasian ethnicity, had naturally dark, untreated hair, reported no use of dyes or chemical straightening, and were not receiving any regular medication at the time of sampling. These criteria followed the quality-control framework adopted in our previous paediatric biomonitoring study of silver but were independently implemented here for a broader elemental suite [33].

Hair collection took place during April–May 2001 as part of the University of Alcalá paediatric exposure programme [32], providing one of the earliest datasets on REEs and technology-related metals in Spain. This historical baseline predates the widespread diffusion of smartphones, nanomaterials, and electric-mobility technologies, and therefore represents pre-industrial reference levels for the study area.

The investigation complied with the Declaration of Helsinki. Written informed consent was obtained from parents or legal guardians of all participants. The protocol used and described here in detail was approved by the *Comité de Ética de la Investigación y de Experimentación Animal* of the University of Alcalá (CEI-EA code CEIP/2025/3/089).

Hair was cut from the occipital region close to the scalp using stainless-steel scissors previously decontaminated with ultrapure water (Milli-Q^®^ Direct 8, Merck Millipore, Darmstadt, Germany; resistivity 18.2 MΩ cm). Each participant contributed approximately 1 g of hair, of which the proximal 1 cm was retained to represent recent exposure, assuming an average growth rate of about 1 cm month^−1^ [34]. Samples were stored in pre-cleaned polyethylene bags, labelled, and transported to the analytical laboratory in acid-washed polypropylene containers.

**Figure 1:**
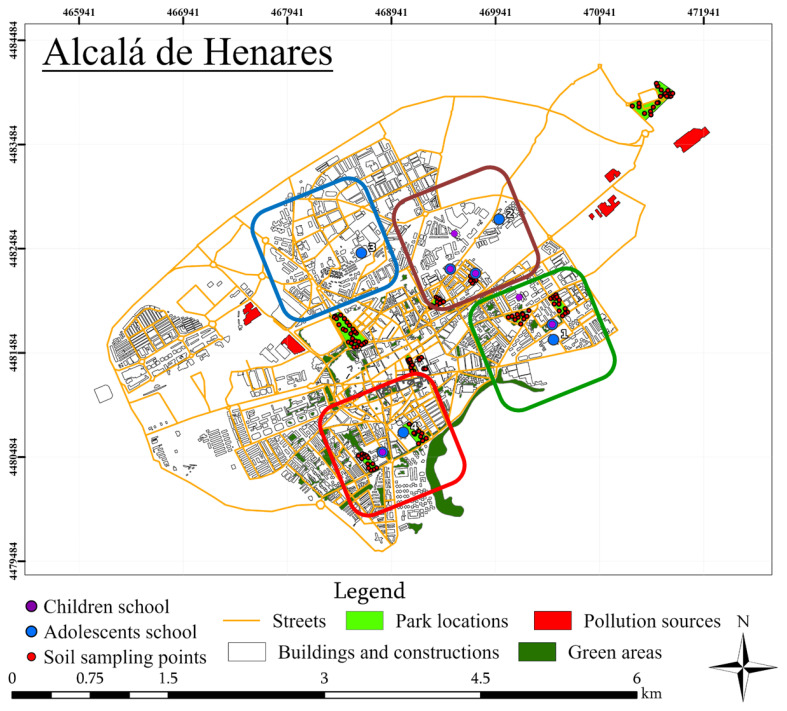
Study area and sampling sites. Children’s primary schools (purple symbols) and adolescents’ secondary schools (blue symbols) are indicated, together with soil sampling locations (red symbols). Coloured outlines delineate the four environmental zones used to evaluate residential settings: Zone I (green outline), low-density housing with abundant green space; Zone II (brown outline), compact urban district with dense residential buildings; Zone III (blue outline), heavy traffic and major roads; and Zone IV (red outline), mixed industrial facilities. Children were recruited from primary schools located in Zones I, II and IV, whereas adolescents were enrolled from secondary schools distributed across all four zones.

To evaluate the influence of residential setting, Alcalá was divided into four environmental zones consistent with prior geochemical and biomonitoring surveys (Figure 1; [33]): Zone I, characterised by low-density housing and abundant green space; Zone II, a compact urban district with dense residential buildings; Zone III, an area dominated by heavy traffic and major roads; and Zone IV, containing mixed industrial facilities. Children were recruited from primary schools located in Zones I, II, and IV, whereas adolescents were enrolled from secondary schools distributed across all four zones. This stratified sampling enabled spatial comparisons of exposure patterns within the municipality.

### 2.3. Pre-Treatment and Analytical Methods

Each scalp hair sample (approx. 1 g) was initially washed to minimise external contamination while preserving the integrity of endogenous trace-element signatures. Samples were immersed for five minutes in a 1% (*v*/*v*) solution of the non-ionic surfactant Triton X-100 (Sigma-Aldrich, St. Louis, MO, USA) prepared with ultrapure Milli-Q^®^ water and sonicated in an ultrasonic bath (J.P. Selecta Ultrasons, Abrera, Barcelona, Spain). This process was repeated four times. After this step, each specimen was rinsed three times with ultrapure water to remove detergent residues and again sonicated for five minutes in ultrapure water alone. The samples were finally rinsed, blotted on acid-washed filter paper, and oven-dried at 50 °C to constant weight [35].

Approximately 100 mg of each cleaned and dried subsample was transferred into acid-washed polytetrafluoroethylene digestion tubes and treated with 2 mL of 65% HNO_3_ (Suprapur^®^, Merck, Darmstadt, Germany) for eight hours at room temperature to allow pre-oxidation. Samples were then heated at 96 °C for 12 h on a programmable digestion block. After cooling, digests were quantitatively diluted to 10 mL with ultrapure water and stored at −80 °C until instrumental analysis. Procedural blanks accompanied every batch of five samples. This digestion procedure was adapted from previously validated hair biomonitoring protocols (e.g., [33,35]) with minor modifications (sample mass and batch blank frequency) to match the present multi-element ICP–MS workflow and available subsample mass.

Elemental quantification of Bi, Ce, Er, Gd, Ir, La, Nd, Pd, Pr, Pt, Rb, Rh, Sb, Th, U, and Y was performed using ICP-MS on a PerkinElmer NexION 350D instrument (Waltham, MA, USA) operated in collision mode with helium gas to minimise polyatomic interferences. Analytical parameters were adapted from validated multi-element biomonitoring protocols to optimise sensitivity for REEs and emerging trace metals [33].

Digested hair solutions were diluted 1:5 with ultrapure Milli-Q^®^ water acidified to 1% HNO_3_ (*v*/*v*). External calibration was performed in a matched acid matrix (1% HNO_3_) using a reagent blank and four calibration standards prepared from certified commercial stock solutions. Calibration curves consisted of a blank and four certified multi-element standards (TraceCERT^®^, Merck, Germany) prepared in the same acid matrix. Calibration linearity was assessed for each analyte, and curves were accepted when they met predefined linearity criteria (R^2^ ≥ 0.995) and when back-calculated standard concentrations were within predefined limits (±10% of nominal values, and ±15% at the lowest calibration level). The external calibration working ranges (ng mL^−1^; i.e., µg L^−1^ in the diluted digest solutions) were 0–11.07 for REEs/Y (La, Ce, Pr, Nd, Gd, Er, and Y), 0–113.06 for Rb, 0–88.09 for Th and U, 0–126.95 for Pd and Pt, 0–126.95 for Rh, and 0–126.95 for Sb. Niobium (Nb) was used as the sole internal standard, introduced continuously to correct for instrumental drift and matrix effects. Each sample digest was analysed in duplicate, with three replicate readings and an integration time of 1 s per isotope (^209^Bi, ^140^Ce, ^167^Er, ^158^Gd, ^193^Ir, ^139^La, ^143^Nd, ^105^Pd, ^141^Pr, ^195^Pt, ^85^Rb, ^103^Rh, ^121^Sb, ^232^Th, ^238^U, ^89^Y).

Quality assurance was verified using two certified reference materials (CRMs). The NCS DC 73347 human-hair CRM (National Research Centre for Certified Reference Materials (CRMs), No. 7 District 11, Hiepjinge 100013, Beijing, China) was analysed every five samples to verify accuracy. Certified concentrations (in µg g^−1^) for selected analytes were: Bi = 0.34 ± 0.02, Ce = 0.12 ± 0.03, La = 0.049 ± 0.011, Sb = 0.095 ± 0.016, and Y = 0.084 ± 0.020. For elements not certified in the hair CRM, we used the SPS-SW2 surface-water CRM (Spectrapure Standards AS, Oslo, Norway; batch 127) as an aqueous multi-element standard. Certified concentrations (in ng mL^−1^ at 20 °C) for the monitored elements were: Er = 2.50 ± 0.02, Gd = 2.50 ± 0.02, Nd = 2.50 ± 0.02, Pr = 2.50 ± 0.02, Rb = 50.0 ± 0.3, Th = 2.50 ± 0.02, and U = 2.50 ± 0.02.

Recoveries for all analytes were within 90–110% of the certified values. Procedural blanks and calibration-verification standards were analysed at the beginning and end of each sequence. Analytical limits of detection (LoD) were defined as 3σ of blank signals for children and adolescents. Minor differences in the calculated limits of detection were attributed to variations in sample mass and dilution volume rather than to analytical performance. Although identical ICP-MS conditions were applied to all specimens, the smaller amounts of hair available after washing and trimming in the adolescent group required higher dilution, which in turn slightly increased the instrumental LoD.

Because trace-element concentrations in keratinised matrices are chemically stable under dry storage, and because all samples underwent the same decontamination and QA/QC-controlled ICP–MS workflow, the dataset provides a defensible historical baseline for pre-diffusion urban exposure.

### 2.4. Rare Earth Element (REE/REY) Normalisation and Anomaly Calculations

REE abundances (Ce, La, Pr, Nd, Gd, and Er, and Y in children) were visualised and interpreted using shale-normalised REE/REY patterns to remove the Oddo–Harkins effect and facilitate comparison of fractionation trends and element-specific decoupling [36,37]. Consistent with recommendations for European environmental and biological samples, concentrations were normalised to the European Shale (EUS) dataset [38]. In addition, sensitivity normalisations were performed using Post-Archean Australian Shale (PAAS; [39]) and the World Shale (WSH) composite [37,38].

For each element *X* and reference set “ref” (EUS, PAAS, or WSH), the normalised concentration was calculated as follows:

*X_N_*_,ref_ = [*X*]_sample_/[*X*]_ref_

Normalised REE patterns were plotted against increasing atomic number using a logarithmic y-axis.

Element anomalies were quantified using the X/X* framework, where *X_N_*_,ref_ is the measured normalised abundance and X*_N__,ref_ is the theoretical value expected for a smooth REE pattern.

Following recent guidance showing that linear interpolations may yield biassed or even aberrant La and Ce anomalies depending on the normalisation used, La* and Ce* were computed by geometric (semi-log) extrapolation from Pr and Nd [40]. Specifically:

La*_N__,ref_ = (Pr*_N_*_,ref_)^3^/(Nd*_N_*_,ref_)^2^

Ce*_N__,ref_ = (Pr*_N_*_,ref_ ^2^)/Nd*_N_*_,ref_

La anomaly: La/La* = La*_N_*_,ref_/La*_N__,ref_ = La*_N_*_,ref_ × (Nd*_N_*_,ref_)^2^/(Pr*_N_*_,ref_)^3^

Ce anomaly: Ce/Ce* = Ce*_N_*_,ref_/Ce*_N__,ref_ = Ce*_N_*_,ref_ × Nd*_N_*_,ref_/(Pr*_N_*_,ref_)^2^

Handling of left-censoring for normalised abundances and anomalies. Because shale-normalised abundances (*X_N_*_,ref_) and anomaly ratios (La/La* and Ce/Ce*) are ratio-based quantities commonly displayed on a logarithmic axis, values below the analytical LoD were not set to zero or substituted, because substitution can bias summary statistics and distort relationships in left-censored datasets [41,42,43]. Instead, for REE-pattern plotting and for the distributional summaries of *X_N_*_,ref_ and anomalies reported in the Supplementary Tables, we applied a strict-valid subset approach: *X_N_*_,ref_ was treated as missing when [*X*]_sample_ was <LoD for that element, and anomalies were computed only when La, Ce, Pr, and Nd were all quantified (non-censored) in the same sample. This conservative restriction prevents mathematically unstable ratios and reduces the risk of artefactual La-Ce anomalies in REE-poor biological samples, consistent with practical guidance for REE normalisation and anomaly calculation [37,40], while concentration-level statistics elsewhere in the manuscript were handled using dedicated left-censored methods (Section 2.5).

### 2.5. Statistical Analysis

Data below the LoD were handled using methods for left-censored environmental datasets, following established guidance [42,44]. When the proportion of censored observations was <50%, summary statistics were derived using the Kaplan–Meier estimator. For datasets with 50–80% censored values, robust regression on order statistics (ROS) was applied when the sample size was <50, and maximum likelihood estimation (MLE) when n > 50. For variables with >80% censoring, only upper percentiles were reported rather than full distributional summaries. Descriptive statistics included arithmetic and geometric means, medians, interquartile ranges (IQR), 95th percentiles, and distribution-based reference values, defined as the 95th percentile together with its 95% confidence interval (CI-PP95), in line with IUPAC recommendations for population reference intervals [45].

Group comparisons by sex and age were carried out using Wilcoxon rank-sum tests or Welch’s *t*-tests, depending on distributional characteristics and homoscedasticity. Spatial differences among residential zones were evaluated using procedures appropriate for censored and uncensored data. For variables containing values below the LoD, Kaplan–Meier estimation was used for zone-level summaries, and an overall Peto–Peto (modified Gehan–Wilcoxon) one-factor test was applied to compare zones [46,47]. Where the overall test indicated differences, post hoc pairwise comparisons between zones were conducted within the same censored-data framework, and *p*-values were adjusted for multiple testing as described below. For fully uncensored variables, test selection was based on diagnostic checks: when normality (Shapiro–Wilk test) and homogeneity of variances (Fligner–Killeen test) were both satisfied, ANOVA followed by Duncan’s multiple range test was applied; when normality was not rejected but variances were unequal, Welch’s ANOVA with appropriate post hoc tests was used; and when normality was rejected, pairwise Wilcoxon rank-sum tests were performed. Across-zone multiple comparisons (and multi-element testing), *p*-values were adjusted using the Benjamini–Hochberg false discovery rate procedure.

To characterise background exposure in this population, reference intervals were derived separately for children and adolescents (and stratified by sex where sample size allowed) by estimating the 95th population percentile (P95) of hair concentrations and its 95% confidence interval (CI-PP95) within the censored-data framework described above. These values are interpreted as upper population reference limits rather than health-based guidance thresholds; throughout the manuscript, the term “reference interval” refers specifically to these P95/CI-PP95 estimates.

To explore potential environmental contributions, non-parametric Spearman rank correlation coefficients (*ρ*) were calculated between hair concentrations and co-located topsoil levels for elements measured in both matrices, namely Sb, Rh, Pt, and Y. Topsoil concentrations for the Alcalá de Henares area were obtained from Peña-Fernández et al. [31] and used here for research purposes. This approach is appropriate for the right-skewed and non-normal distribution of environmental measurements. Statistical significance was set at *α* = 0.05. All analyses were performed in R (version 4.4.3; [48]) using the *NADA2* and *rstatix* packages.

## 3. Results

### 3.1. Detection Frequencies and Concentrations in Children

Hair samples from 120 children were analysed for seventeen elements, revealing distinct detection patterns between REEs and emerging metals (Table 1, Table 2 and Table 3, for all children and by sex). Within the REE suite, Ce, Nd, Pr, Gd, and Er were the most frequently quantified, while Y was not detected, and La showed the highest proportion of censored results among the detected REEs. Detection frequencies were therefore heterogeneous across the REE suite rather than showing a simple light vs. heavy predominance. Median concentrations ranged from 0.00035 µg g^−1^ (Er) to 0.011 µg g^−1^ (Ce), with Ce and La showing the highest values. The 95th percentiles reached 0.031 µg g^−1^ for Ce and 0.022 µg g^−1^ for La. Accordingly, the REEs ordered by median concentration (Table 1) were (µg g^−1^): Ce (0.011) > La (0.007) > Nd (0.004) > Pr (0.001) > Gd (0.0007) > Er (0.0004). Given the relatively good detection frequencies in this age group, the median provides a robust summary of the REE profile in children.

For REE pattern-shape evaluation, shale-normalised REE patterns and La/Ce anomaly summaries are provided in the Supplementary Information (Figures S1 and S2; Tables S1 and S2).

**Figure 2:**
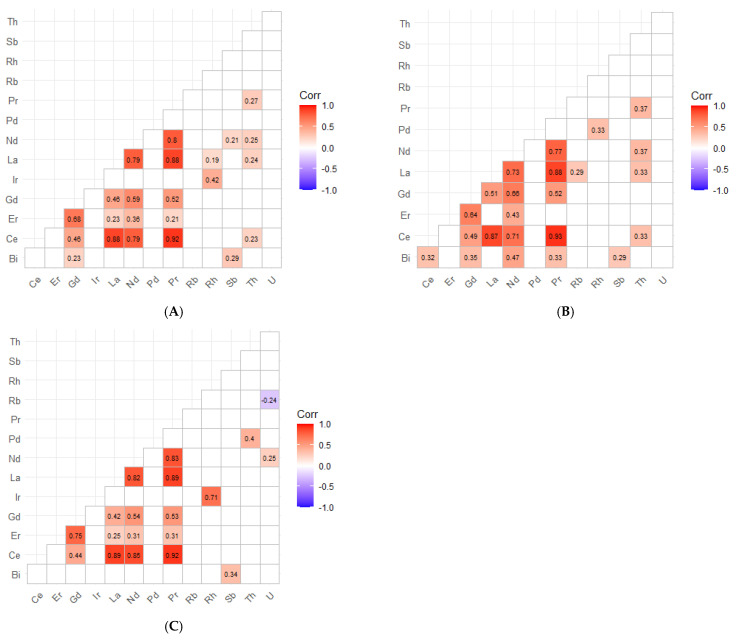
Inter-element correlations in children’s hair (overall and by sex) from Alcalá de Henares, Spain. Heatmaps show Spearman rank correlation coefficients (ρ) between hair element concentrations for (**A**) all children, (**B**) boys, and (**C**) girls. Warmer colours indicate positive correlations, and cooler colours indicate negative correlations (scale −1 to +1). Correlations were calculated using pairwise complete observations; therefore, the number of observations varies across element pairs. Statistical significance was assessed at a two-sided *p*-value < 0.05 after Benjamini–Hochberg false discovery rate (FDR) correction; non-significant correlations are masked (white cells).

Inter-element correlations in children’s hair would suggest a coherent REE mixture. The REEs were strongly and positively inter-correlated, particularly among the light REEs, with high Spearman correlations for Ce-Pr (*ρ* = 0.92), La–Ce (*ρ* = 0.88), La–Pr (*ρ* = 0.88), Nd-Pr (*ρ* = 0.80), La-Nd (*ρ* = 0.79), Er-Gd (*ρ* = 0.68), Gd-Nd (*ρ* = 0.59), Gd-Pr (*ρ* = 0.52), and moderate-to-strong correlations extending from La-Gd (*ρ* = 0.46) and Nd-Er (*ρ* = 0.36) (Figure 2). Sex-stratified heatmaps confirmed that the REE mixture structure was highly consistent in both boys and girls (Figure 2B,C). In boys, light REEs remained strongly inter-correlated (e.g., Ce–Pr *ρ* = 0.93; La–Pr *ρ* = 0.88; La–Ce *ρ* = 0.87; Nd–Pr *ρ* = 0.77; La–Nd *ρ* = 0.73), with similarly strong coupling among the heavier REEs (Er–Gd *ρ* = 0.64; Gd–Nd *ρ* = 0.66). In girls, the same pattern was observed, with high correlations among the light REEs (Ce–Pr *ρ* = 0.92; La–Ce *ρ* = 0.89; La–Pr *ρ* = 0.89; La–Nd *ρ* = 0.82; Nd–Pr *ρ* = 0.83) and moderate-to-strong correlations extending to the heavier REEs (Er–Gd *ρ* = 0.75; Nd–Gd *ρ* = 0.54; Gd-Pr *ρ* = 0.53).

Among the emerging and technology-related elements, Sb and U exhibited the highest detection frequencies and concentrations, being quantified in almost all children’s samples (n = 119 and 120, respectively). Bi was also commonly detected. In contrast, Rb and Pd presented high levels of censored values (>77%), while Rh (n = 6), Th (n = 5), and Ir (n = 1) were measurable only in a few cases, with concentrations near their limits of detection (0.0018–0.006 µg g^−1^). Pt was not detected in any sample (LoD = 0.005 µg g^−1^). In children, the concentrations of the emerging elements, ordered using median values where available and upper-percentile estimates for sparsely detected analytes (µg g^−1^), were: Sb (0.033) > Rb (0.030; n = 27) ≈ Rh (0.030; n = 6) > U (0.011) > Th (0.009; P97.5; n = 5) > Bi (0.006) > Pd (0.001; P95; n = 12). Ir (0.0023) was detected in only one sample, and Pt remained undetected in all samples. In contrast with the REE, inter-element correlations among the emerging elements were generally weak and are reported in Figure 2; interpretation is limited by high censoring for several analytes. After FDR correction (Figure 2), correlations involving the emerging elements were sparse and generally weak. In the full children’s cohort, Bi showed a weak positive association with Sb (*ρ* = 0.29) and Gd (*ρ* = 0.23), while Th showed only low-magnitude correlations with selected REEs (e.g., Pr–Th ρ = 0.27; Nd–Th ρ = 0.25; La–Th ρ = 0.24; Ce–Th ρ = 0.23). Sex-stratified heatmaps indicated heterogeneity: in boys, Bi correlated moderately and positively with Ce/Gd/Nd/Pr and Sb (*ρ* = 0.32–0.47) and Th with La/Ce/Pr/Nd (*ρ* = 0.33–0.37), whereas in girls, U was inversely correlated with Rb (*ρ* = −0.41) and positively with Nd (*ρ* = 0.25), Bi was correlated positively with Sb (ρ = 0.34), and Th with Pd (*ρ* = 0.40). Consistent with this, REE distributions were highly similar by sex at the central tendency level, with near-identical medians in boys vs. girls (Table 2 and Table 3) for Ce (0.0109 vs. 0.0109 µg g^−1^), La (0.00711 vs. 0.00720), Nd (0.00389 vs. 0.00430), Pr (0.0014 vs. 0.0013), Gd (0.00058 vs. 0.00073), and Er (0.00031 vs. 0.00035), and overlapping IQRs across all REEs, although P95 values were generally higher in boys for several REEs (e.g., Ce and La).

Sex-stratified results (Table 4) showed statistically significant sex differences for Sb, Rb, and U. Boys presented higher median Sb concentrations than girls (0.048 vs. 0.027 µg g^−1^) and lower U levels (0.008 vs. 0.014 µg g^−1^), while Rb exhibited a sex-dependent pattern, with higher values in girls, particularly at the upper end of the distribution (97.5th percentile: 0.0191 vs. 0.0089 µg g^−1^). In contrast, differences for the REEs were minimal, with overlapping interquartile ranges across sexes; no statistically significant sex-related differences were observed for any REE in children (Table 4). Among the low-detection analytes, Ir was detected in one girl only (0.002 µg g^−1^); Pd was detected in six participants for each sex; Rh in four boys and two girls; and Th in four boys and one girl. No statistically significant sex-related differences were observed for these low-frequency analytes after adjustment using the Benjamini–Hochberg false-discovery-rate procedure.

### 3.2. Detection Frequencies and Concentrations in Adolescents

Hair samples from adolescents (n = 97) were analysed for sixteen elements, as Y was not measured in this group. Detection patterns varied widely among analytes, with generally lower frequencies and concentrations than in children (Table 5, Table 6 and Table 7).

Within the REE suite, detections were limited. La (n = 8) and Er (n = 8) were the most frequently quantified, followed by Ce (n = 6), Pr (n = 5), Nd (n = 3), and Gd (n = 1). The small number of samples above the detection limit precluded the calculation of medians or IQR [44]. Concentrations in these few positive samples were very low, typically ranging between 0.0004 µg g^−1^ (Er) and 0.098 µg g^−1^ (Ce), and were generally close to the analytical limits of detection. Using the available upper-percentile estimates (P95, P97.5, or, when necessary, the single detected value), the approximate ordering of REEs by high-percentile concentration was (µg g^−1^): Ce (0.021, 0.026) > La (0.012, 0.018) > Nd (0.013; P97.5) > Pr (0.0027, 0.0034) > Gd (single detected value, 0.0022) > Er (0.0005, 0.0008). However, these upper-percentile estimates should be interpreted cautiously as indicative only, reflecting the limited number of samples with concentrations above the detection limit.

**Figure 3:**
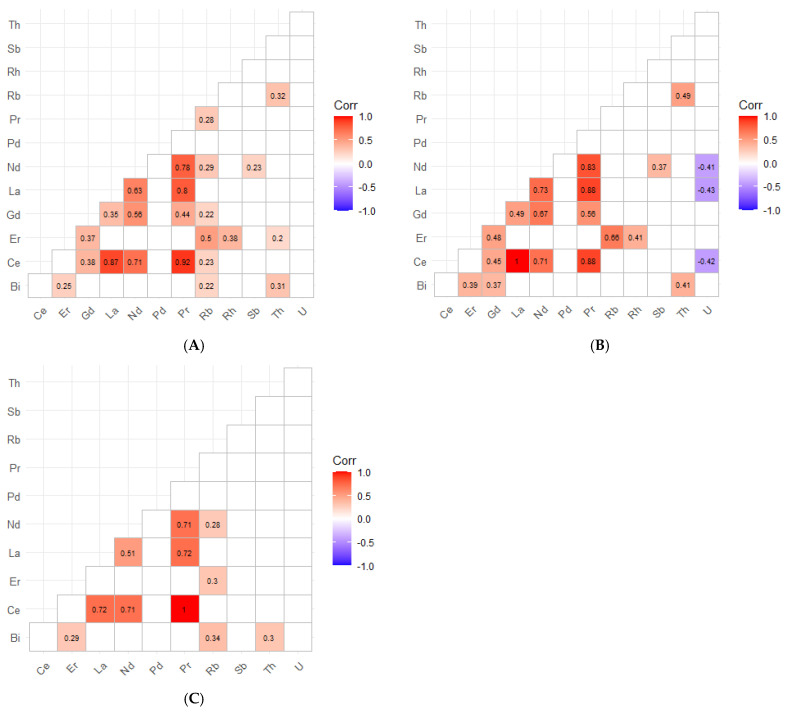
Inter-element correlations in adolescents’ hair (overall and by sex) from Alcalá de Henares, Spain. Heatmaps show Spearman rank correlation coefficients (ρ) between hair element concentrations for (**A**) all adolescents, (**B**) males, and (**C**) females. Warmer colours indicate positive correlations, and cooler colours indicate negative correlations (scale −1 to +1). Correlations were calculated using pairwise complete observations; therefore, the number of observations varies across element pairs. Statistical significance was assessed at a two-sided *p*-value < 0.05 after Benjamini–Hochberg false discovery rate (FDR) correction; non-significant correlations are masked (white cells).

Inter-element correlations in adolescents’ hair also supported a coherent REE mixture where quantifiable (Figure 3). After Benjamini–Hochberg FDR correction, the overall cohort showed strong within-suite coupling among light REEs: Ce correlated strongly with La (*ρ* = 0.87) and Pr (*ρ* = 0.92), La correlated with Nd (*ρ* = 0.63) and Pr (*ρ* = 0.80), and Nd correlated with Pr (*ρ* = 0.78). Additional moderate correlations extended to heavier REEs and co-measured elements (e.g., Gd–Nd *ρ* = 0.56; Er–Rb *ρ* = 0.50), consistent with a structured mixture profile rather than isolated element behaviour (Figure 3A). Sex-stratified analyses showed that this REE structure was most pronounced in males (Ce–La *ρ* = 1.00; Ce–Pr *ρ* = 0.88; La–Pr *ρ* = 0.88; Nd–Pr *ρ* = 0.83; La–Nd *ρ* = 0.73; Ce–Nd *ρ* = 0.71; Gd–Nd *ρ* = 0.67) (Figure 3B). In females, fewer significant REE pairs were retained after FDR correction, but the core light-REE coherence remained evident (Ce–Pr *ρ* = 1.00; Ce–La *ρ* = 0.72; Ce–Nd *ρ* = 0.71; Nd–Pr *ρ* = 0.71; La–Pr *ρ* = 0.72; La–Nd *ρ* = 0.51) (Figure 3C).

Similarly to what was observed in the children’s cohort, Sb (0.009) and U (0.016) showed the highest detection frequencies and concentrations, being quantified in almost all adolescent samples. Th (n = 64) displayed a moderate detection frequency but low concentrations (0.002; all median values in µg g^−1^). Bi (n = 38) and Rb (n = 25) were also detected in a subset of samples, with medians of 0.002 and 0.004 µg g^−1^, respectively. Pd (n = 7) and Rh (n = 6) were measured only sporadically, while Ir and Pt were not detected in any sample, remaining below their detection limits (0.0025 and 0.0063 µg g^−1^, respectively). Overall, most elements were present at trace levels, generally within one order of magnitude above their respective detection limits. In adolescents, the emerging elements, ordered by their median concentrations where available and by typical detected values for sparsely detected analytes (µg g^−1^), were: U (0.016) > Sb (0.009) > Rb (0.004) > Bi (0.002) ≈ Th (0.002) > Pd (0.0015; P95; n = 7) > Rh (0.0005; P95; n = 6), while Ir and Pt consistently remained below their limits of detection (0.0025 and 0.0063 µg g^−1^, respectively).

Correlations involving emerging/technology-related elements were comparatively sparse, consistent with limited detectability for several analytes and the masking of non-significant coefficients in the heatmaps. In the overall adolescent cohort, the few retained cross-element associations included Rb–Th (*ρ* = 0.32), Bi–Th (*ρ* = 0.31), and weak positive correlations between Nd and Sb (*ρ* = 0.23) and between Pr and Rb (*ρ* = 0.28) (Figure 3A). In males, U showed inverse associations with multiple REEs (Ce *ρ* = −0.42; La *ρ* = −0.43; Nd *ρ* = −0.41), while Rb-Er and Rb–Th were positive (*ρ* = 0.68 and 0.49, respectively), and Bi–Th remained positive (*ρ* = 0.41) (Figure 3B). In females, only a small number of non-REE correlations remained significant after FDR (e.g., Bi–Rb *ρ* = 0.34; Bi–Th *ρ* = 0.30; Er–Rb *ρ* = 0.30), and no significant U correlations were observed (Figure 3C).

Sex-stratified results (Table 8) revealed statistically significant differences for Ce, Er, Rb, and U, although the high proportion of censored values for REEs in this cohort should be carefully considered. Male participants exhibited higher upper-percentile concentrations of Ce and Er (P97.5 = 0.047 and 0.0008 µg g^−1^, respectively) compared with girls (0.022 and 0.0006 µg g^−1^), whereas Rb (P97.5 = 0.045 vs. 0.013 µg g^−1^) and U (0.020 vs. 0.007 µg g^−1^) were significantly higher in females. Differences for the remaining elements were minimal, with overlapping interquartile ranges. Among the low-detection analytes, Pd was detected in five female and two male adolescents, while Rh was detected only in six male participants. After adjustment using the Benjamini–Hochberg false-discovery-rate procedure, only the sex differences for Rb and U remained statistically significant.

### 3.3. Age Comparison

Age-related differences were evaluated primarily in terms of detectability and distributional behaviour because censoring differed markedly between children (6–9 years) and adolescents (13–16 years), particularly for REEs. REEs were quantified far more frequently in children, whereas adolescent REE data were dominated by results below the limit of detection; accordingly, age contrasts are best interpreted as differences in detection structure and upper-tail behaviour rather than as simple shifts in central tendency (Table 9). In addition, part of the lower detectability in adolescents may be methodological rather than exposure-related, because the smaller hair masses available required higher dilution and therefore slightly higher instrumental LoDs despite identical ICP-MS conditions.

Age-related differences between children and adolescents were evaluated using the Peto–Peto one-factor test for censored data (Table 9). Overall, children showed substantially higher REE detectability, whereas adolescent REE distributions were strongly left-censored for most analytes; therefore, age contrasts are best interpreted as distributional differences (including detectability and upper-tail behaviour) rather than as simple shifts in central tendency. Consistent with this, Ce differed by age (children median 0.0109 µg g^−1^, IQR 0.0053–0.0175; adolescents P97.5 0.026; *p*-value = 0.0184), La (0.0072, 0.0055–0.0116; adolescents P97.5 0.018; *p*-value = 0.00052), Pr (0.00137, 0.00074–0.00213; adolescents P97.5 0.0034; *p*-value = 0.0215), Nd (0.0042, 0.0028–0.0066; adolescents P97.5 0.013; *p*-value = 0.0374) and Gd (0.00068, 0.00045–0.00102; adolescents 0.00216; *p*-value = 0.0374) differed significantly by age, with adolescents showing higher upper-end values where quantifiable. Er also differed significantly (children median 0.00035, IQR 0.00025–0.00059; adolescents P97.5 0.0008; *p*-value = 8.41 × 10^−7^), again reflecting a contrast expressed primarily in the detectable/upper tail under heavy censoring.

Among emerging/technology-related elements, children showed clearly higher Sb and Bi (Sb: children median 0.033, IQR 0.017–0.068; adolescents median 0.0092, IQR 0.006–0.016; *p*-value = 2.14 × 10^−22^; Bi: children median 0.006, IQR 0.005–0.016; adolescents median 0.002, IQR 0.001–0.005; *p*-value = 1.8 × 10^−6^). Rh also differed (children median 0.030, IQR 0.022–0.037; adolescents P97.5 0.00006; *p*-value = 0.026), but this contrast should be interpreted cautiously given sparse detection near reporting limits. Pd showed a modest age effect expressed in the upper tail (children P97.5 0.00111 vs. adolescents P97.5 0.0016; *p*-value = 0.0424). By contrast, Rb and U did not differ significantly by age (Rb: children median 0.0298, IQR 0.0225–0.0373; adolescents median 0.004, IQR 0.001–0.012; *p*-value = 0.303; U: children median 0.011, IQR 0.007–0.020; adolescents median 0.016, IQR 0.005–0.030; *p*-value = 0.549). Th also showed no significant age difference (children P97.5 0.0085 vs. adolescents median 0.002, IQR 0.001–0.003; *p*-value = 0.498), but this should be interpreted cautiously given the strong censoring and the fact that the hypothesis test did not support a distributional difference.

Finally, given the right-skewed nature of several distributions and the extent of left-censoring (especially in adolescents), the observed age patterns likely reflect a combination of detectability thresholds and a small number of higher-exposure individuals driving the upper tail, rather than uniform shifts across the full concentration distributions.

### 3.4. Shale-Normalised REE Patterns and La/Ce Anomalies

Shale-normalised REE patterns were evaluated using three reference composites (EUS, PAAS, and WSH) to facilitate comparison of pattern shape independently of absolute concentrations. Median patterns were broadly subparallel across normalisers, indicating that the qualitative fractionation profile is not materially influenced by the selected reference composite (Figure S1).

Lanthanum and cerium anomalies (La/La* and Ce/Ce*) calculated on shale-normalised values showed consistent central tendencies across normalisers, with dispersion primarily driven by the upper tail (Figure S2; Tables S1 and S2). Anomaly summaries refer to the strict-valid subset defined in Section 2.4 (i.e., computed only when La, Ce, Pr, and Nd were quantified within the same sample). Across both cohorts and all normalisers, anomaly distributions were centred below unity, with dispersion driven primarily by the upper tail and occasional high-end values in a small subset of samples. Given the trace-level REE context and the ratio-based nature of anomaly metrics, values below 1 are most parsimoniously interpreted as the absence of positive La/Ce anomalies rather than evidence of systematic fractionation; at very low concentrations, modest analytical variability in the Pr–Nd interpolation framework can translate into downward-shifted ratio distributions even when concentration profiles are geochemically featureless. This would support a conservative interpretation of anomalies in hair as descriptive pattern-shape indicators rather than as strong source-diagnostic signals in this low-loading urban baseline.

### 3.5. Residential Areas

Table 10 and Table 11 present zone-specific distributions (and corresponding statistical comparisons) of hair REEs and emerging trace elements in children and adolescents, respectively, across the defined residential areas of Alcalá de Henares (Zone I: low-density housing/green space; Zone II: compact, dense urban district; Zone III: heavy-traffic/major-road corridor; Zone IV: mixed industrial facilities). Children were sampled in Zones I, II and IV, whereas adolescents were sampled across all four zones. Values are reported as Kaplan–Meier (KM) means, estimated from the empirical survival function to account for left-censored results (<LoD), which provides a more robust basis for group comparisons than the arithmetic mean in censored datasets.

Statistically significant between-zone differences were observed for Ce (*p*-value = 0.005), Er (*p*-value = 1.04 × 10^−10^), Gd (*p*-value = 0.004), and Pr (*p*-value = 0.012) in children’s hair. Specifically, Ce was higher in the more urban/industrial zones (Zone II and Zone IV) than in the greener low-density area (Zone I) (KM means: 0.015–0.016 vs. 0.008 µg/g). In contrast, Er showed the opposite pattern, with the highest values in Zone I (0.00073 µg/g) and markedly lower values in Zones II–IV (0.00034–0.00038 µg/g). Gd was also highest in Zone I (0.0012 µg/g), lower in Zone II (0.0007 µg/g), and intermediate in Zone IV (0.0009 µg/g). Pr showed its highest values in Zone IV (0.0020 µg/g), exceeding Zones I–II (0.0013–0.0016 µg/g). For the remaining analytes in children (Bi, La, Li, Nd, Pd, Rb, Sb, and U), no statistically significant zonal differences were detected (all *p* > 0.05), although several showed modest numerical shifts (e.g., Sb and U tending to higher KM means in Zone IV).

The clearest zonal separation in adolescents occurred for Rb (*p*-value = 3.64 × 10^−22^), driven by markedly elevated values in Zone IV (KM mean 0.050 µg/g) compared with Zones I–III (0.013–0.016 µg/g). Sb also differed by zone (*p*-value = 0.0096), with the highest KM mean in Zone II (0.019 µg/g), lower levels in Zones I and III (0.010–0.011 µg/g), and Zone IV showing an intermediate pattern (0.013 µg/g). Th showed a significant overall zone effect (*p*-value = 0.0056), with KM means slightly higher in Zones II/IV (both 0.0037 µg/g) than in Zones I/III (0.0022–0.0023 µg/g), and post hoc grouping indicating separation involving Zone IV. By contrast, Bi and U did not differ significantly across zones (*p*-values = 0.490 and 0.138, respectively), although U exhibited a numerically higher KM mean in Zone III (0.080 µg/g), consistent with a small upper tail rather than a consistent zone shift.

### 3.6. Soil–Hair Associations

**Figure 4:**
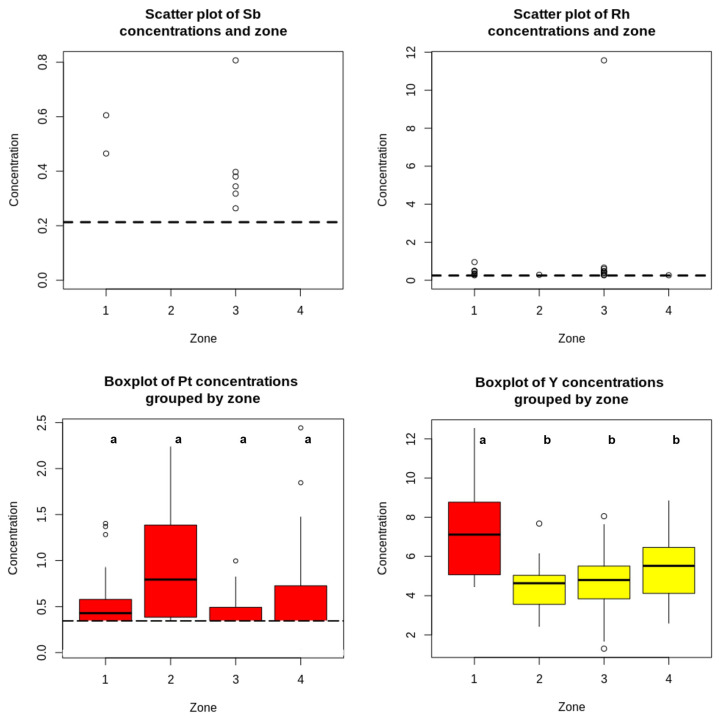
Levels of Sb, Rh, Pt, and Y in topsoils monitored in each main area in Alcalá de Henares. Box-and-whisker plots for Pt and Y concentrations (mg/kg). The line inside the box represents the median value; the boxes mark the 25th and 75th percentiles; the horizontal lines outside the box (whiskers) mark the values that extend 1.5 times the width of the box. Points outside the whisker are called outliers, and these are marked with an extreme symbol if they are more than three times the width of the box. The concentration values (arithmetic mean (µg/g) ± SD) with different letters were significantly different (*p*-value = 0.0321 and 0.000044, respectively). Colours represent statistically distinct groups based on the post hoc multiple-comparison lettering; groups sharing the same letter are not significantly different.

To aid interpretation of potential environmental contributions, spatial patterns in topsoil were examined across the four residential zones. Topsoil concentrations of Sb, Rh, Pt, and Y showed heterogeneous spatial structuring by zone (Figure 4; Table S3). For Sb and Rh, distributions were dominated by left-censoring and near-reporting-limit values; therefore, scatter plots are presented, as boxplot summaries become uninformative when a large fraction of observations are non-detects. Sb showed a patchy pattern, with quantifiable values primarily in Zones I and III (higher central tendency in Zone III; median 0.117 mg kg^−1^, IQR 0.063–0.218), while Zones II and IV were largely non-detect (LoD = 0.213). Rh also exhibited substantial censoring but showed measurable values in Zones I and III (medians 0.172 and 0.185 mg kg^−1^, respectively), with only upper-percentile estimates in Zones II and IV, indicating more localised variability rather than a consistent municipality-wide gradient. In contrast, Pt and Y could be summarised using box-and-whisker plots. Pt concentrations were generally low and right-skewed, and although an overall zone effect was detected (*p*-value = 0.0321), post hoc grouping did not resolve clear pairwise differences after multiple-comparison control (shared letter code). Y showed the clearest zonal contrast (*p*-value = 0.000044), with Zone I exhibiting higher concentrations (median 7.111 mg kg^−1^; IQR 5.071–8.770) than Zones II–IV (medians 4.638–5.514 mg kg^−1^), consistent with a robust zone-level gradient.

Accordingly, subsequent soil–hair analyses focused on whether these zone-level soil patterns translated into variation in internal burden, using hair concentrations as a biomarker. Because zonal soil data were available only for Sb, Rh, Pt, and Y (Figure 4), and because Pt was not detected in hair in either cohort and Y was not detected in children (and was not assessed in adolescents), soil–hair concordance could be evaluated meaningfully only for Sb and Rh. Thus, Spearman rank correlations were therefore computed between hair Sb (Sb_h) and hair Rh (Rh_h) and the corresponding zone-level topsoil concentrations. These correlations are presented for the overall cohorts and stratified by sex to assess potential effect modification by sex while retaining the ecological nature of the soil metric (zone-level soil summaries).

In children, soil–hair correlations were weak overall. In the full cohort, Sb_h showed no meaningful association with soil Pt (*ρ* = 0.04), soil Rh (*ρ* = −0.10), or soil Sb (*ρ* = −0.10), while Rh_h showed small correlations with soil Pt (*ρ* = −0.07) and soil Sb (*ρ* = −0.11) and a weak inverse association with soil Rh (*ρ* = −0.25; *p*-value < 0.01). Sex-stratified analyses were consistent with limited correspondence: in girls, coefficients remained close to zero for Sb_h vs. soil Pt/Rh/Sb (*ρ* = 0.11, −0.09, −0.13, respectively) and Rh_h vs. soil Pt/Rh/Sb (*ρ* = −0.10, −0.23, −0.09), whereas in boys Sb_h vs. soil Pt/Rh/Sb remained weak (*ρ* = −0.05, −0.12, −0.05) and Rh_h showed a modest inverse correlation only with soil Rh (*ρ* = −0.29; *p*-value < 0.05), with non-significant associations for Rh_h vs. soil Pt (*ρ* = −0.06) and Rh_h vs. soil Sb (*ρ* = −0.15).

In adolescents, soil–hair associations were more frequently observed than in children but remained modest in magnitude. In the overall cohort, Sb_h correlated positively with soil Pt (*ρ* = 0.29, *p* < 0.01) and inversely with soil Rh (*ρ* = −0.22, *p* < 0.05) and soil Sb (*ρ* = −0.26, *p* < 0.05). Rh_h showed weak correlations with soil Pt (*ρ* = 0.14) and soil Sb (*ρ* = −0.17) and a weak inverse association with soil Rh (*ρ* = −0.20, *p* < 0.05). Sex-stratified analyses indicated that these patterns were more evident in females. In adolescent girls, Sb_h showed the clearest soil-related signal, with a positive association with soil Pt (*ρ* = 0.33, *p* < 0.01) and inverse associations with soil Rh (*ρ* = −0.30, *p* < 0.05) and soil Sb (*ρ* = −0.32, *p* < 0.01). Rh_h was not detected in female adolescents, precluding sex-specific evaluation for this element. In adolescent males, soil–hair correlations were weak and largely non-significant: Sb_h showed no meaningful association with soil Pt/Rh/Sb (*ρ* = −0.05, −0.12, and −0.05, respectively). In contrast, Rh_h exhibited a weak inverse association with soil Rh (*ρ* = −0.29, *p* < 0.05), while correlations with soil Pt (*ρ* = −0.06) and soil Sb (*ρ* = −0.15) were not statistically significant.

Collectively, these results indicate that zone-level topsoil gradients in Sb, Rh, and Pt are not a dominant determinant of hair Sb or Rh in either children or adolescents from Alcalá de Henares. Where statistically significant associations were observed, they were weak to modest in magnitude and sex-dependent, with the most reproducible pattern being a weak positive association between Sb_h and soil Pt, particularly among adolescent females, alongside generally weak inverse associations with soil Sb and/or soil Rh. Overall, the predominance of small coefficients and the absence of consistent Sb_h–soil Sb and Rh_h–soil Rh correspondence suggest that other exposure pathways (for example, inhalation of traffic-related aerosols and resuspended dust, indoor microenvironments, and dietary intake and consumer-product sources) are likely to contribute more substantially to inter-individual variability in hair burdens than direct soil contact alone.

### 3.7. Reference Values

Proposed reference intervals for children are presented in Table 1, Table 2 and Table 3 for the total group and stratified by sex. For REEs, the 95th percentile reference intervals (CI-PP95, µg g^−1^) were as follows: Ce, 0.0231–0.0414; La, 0.0145–0.0277; Nd, 0.0081–0.0126; Pr, 0.0030–0.0046; Gd, 0.0014–0.0023; and Er, 0.0007–0.0013. Y was not detected in any child and could therefore not be characterised. Among the emerging and technology-related elements, reference intervals were observed for Bi (0.023–0.172 µg g^−1^), Rb (0.046–0.078 µg g^−1^), Rh (0.046–0.078 µg g^−1^), Sb (0.115–0.309 µg g^−1^), and U (0.037–0.077 µg g^−1^). Pd and Th exhibited much lower upper percentiles (P95 and P97.5 of 0.00096 and 0.0085 µg g^−1^, respectively), reflecting their limited detection and low concentrations, while Ir and Pt could not be characterised because they were not detected in any child. For Rb, Sb, and U, which showed sex-dependent patterns, sex-specific reference intervals are recommended. In boys, the CI-PP95 (all in µg g^−1^) was 0.0182 µg g^−1^ for Rb (95th percentile value), 0.127–0.301 for Sb, and 0.020–0.054 for U. In girls, the corresponding values were 0.019–0.031 for Rb, 0.083–0.389 for Sb, and 0.038–0.291 for U, respectively.

For adolescents, proposed reference intervals are summarised in Table 5, Table 6 and Table 7 for the overall cohort and by sex. Owing to the high proportion of censored data, robust 95th percentile estimates could only be derived for a subset of REEs. For Ce, La, and Er, the P95 values were 0.021, 0.012, and 0.0005 µg g^−1^, respectively, and are used as distribution-based upper reference limits for this group. For Nd and Pr, only percentiles (P97.5 = 0.013 and P95 = 0.0027 µg g^−1^, respectively) could be estimated; these are reported as indicative upper bounds rather than formal reference intervals. Gd was detected in only one sample and could therefore not be characterised.

For the emerging elements, Bi, Rb, Sb, Th, and U provided more stable reference estimates. The P95 (CI-PP95; all in µg g^−1^) values were 0.018 for Bi (0.012–0.029), 0.066 for Rb (0.036–0.122), 0.039 for Sb (0.026–0.047), 0.013 for Th (0.003–0.019), and 0.140 for U (0.063–0.212). Pd and Rh showed lower P95 values close to their limits of detection (0.0015 and 0.0005 µg g^−1^, respectively), indicating that most adolescents had only trace-level exposure, while Ir and Pt remained below detection in all adolescent samples. For elements that displayed sex-related differences (in particular Ce, La, Er, Rb, and U), sex-specific P95 or CI-PP95 estimates are provided in Table 6 and Table 7 when available, and should be used when defining reference intervals by biological sex.

## 4. Discussion

Interpretation of element concentrations in hair requires caution because hair reflects both endogenous incorporation during growth (via the follicle’s blood supply) and potential exogenous contributions from adhered particles and surface contamination (e.g., dust, personal-care products, adsorption/desorption processes). This is particularly relevant for trace elements present at low concentrations and in settings with heterogeneous particulate loading. In this study, we applied a robust, standardised decontamination protocol and interpreted the results primarily as biomonitoring indicators of relative exposure. The use of non-ionic surfactants such as Triton X-100 is well established in biomonitoring because these agents efficiently remove exogenous particulate material while minimising alteration of the hair matrix and leaching of endogenous metals [14,32]. Although external deposition can contribute to measured trace metals and REEs—especially under high-pollution outdoor conditions—evidence indicates that effective washing protocols substantially reduce this potential bias, strengthening the interpretability of hair measurements for exposure assessment [14]. Confidence is greatest where signals are coherent at the mixture level (e.g., consistent REE co-variation) and where patterns are consistent with independent environmental gradients, recognising that hair is not an equivalent proxy of internal dose for all elements.

### 4.1. Rare Earth Elements (REEs) in Hair: Children and Adolescents

In this paediatric–adolescent cohort from Alcalá de Henares, the REE profile in hair is characterised by (i) low absolute concentrations and (ii) a coherent within-suite “mixture structure”, with strong positive inter-element associations within the light REEs and extending towards mid/heavier REEs. This coherence is a recurrent feature of REE biomonitoring and is consistent with shared upstream drivers (co-emission, shared exposure pathways, and broadly similar geochemical behaviour), even when matrices differ (hair vs. blood/serum). In Spain and Europe, however, direct paediatric hair datasets for REEs remain limited, and much of the available evidence derives from internal matrices (e.g., cord blood, serum), complicating strict matrix-to-matrix comparisons [10,11]. Accordingly, the present dataset provides novel baseline information for a European youth setting and supports the use of hair as a pragmatic, low-burden matrix for surveillance when venous sampling is not feasible [49].

Although hair and internal matrices capture different exposure windows and are not directly interconvertible, Spanish/European biomonitoring studies provide useful contextual anchors for the magnitude and detectability of La and Ce in populations with broadly comparable environmental settings. In Spanish cord blood, Cabrera-Rodríguez et al. [11] quantified 44 elements and reported median concentrations of approximately Ce 0.030 ng/mL and La 0.010 ng/mL (i.e., 30 and 10 ng/L). These values illustrate that, even in an internal matrix reflecting prenatal exposure, La and Ce are typically present at low ng/L levels in a European setting.

Similarly, in a well-controlled study of lactating mothers (Madrid vs. Munich), Höllriegl et al. [50] reported cerium in Spanish serum spanning 21.6–70.3 ng/L (median 40.4 ng/L), whereas many German plasma/serum samples were near or below a 10 ng/L quantification limit. This cross-country contrast would support the interpretation that background Ce in internal matrices can be close to quantification limits, and that moderate differences between European populations may be measurable when QA/QC is robust.

Notably, internal-matrix concentrations can vary substantially by sample type and study context. In a Canary Islands case–control study (whole blood), Medina-Estévez et al. [51] reported higher values in controls, including Ce 18.0 ng/mL (and 15.0 ng/mL in cases), alongside La 0.011 ng/mL. The much higher magnitude for Ce relative to serum/plasma studies highlights the importance of matrix comparability (whole blood vs. serum/plasma), unit harmonisation, and analytical context when drawing cross-study contrasts. Accordingly, these data are best used as evidence that La/Ce are measurable in Spanish internal matrices and can show considerable variability, rather than as strict numeric comparators to hair.

A broader European anchor is provided by the Andalusian general-population plasma biomonitoring study by Henríquez-Hernández et al. [52], which measured 45 inorganic elements, including REEs, in several hundred participants and reported detectable REEs with covariate associations (e.g., BMI with cerium).

Together, these Spanish datasets indicate that La and Ce are quantifiable in European populations across internal matrices, but commonly at low levels (often close to method limits in plasma/serum), supporting careful interpretation of ratio-based metrics derived from trace-level data.

While these internal-matrix values are not directly comparable to hair, they provide a European exposure context for La/Ce and highlight that internal concentrations can be close to method limits in general-population settings.

Across cohorts, the magnitudes observed in Alcalá are most consistent with low-level background/urban exposure rather than any high-exposure scenario. In children, median concentrations for the most frequently quantified REEs were in the low ng g^−1^ range (e.g., Ce ≈ 0.011 µg g^−1^; La ≈ 0.007 µg g^−1^; Nd ≈ 0.004 µg g^−1^; Pr ≈ 0.001 µg g^−1^), with a stable within-suite correlation structure. Adolescents displayed a broadly similar REE mixture pattern, but with concentrations generally low and frequently close to reporting limits, consistent with modest environmental loading and heterogeneous low-intensity exposures rather than a dominant point source.

Available European reference evidence supports this interpretation. In a non-occupationally exposed urban population from north-east Sweden (aged 1–76 years), median La and Ce in hair were approximately 0.018–0.019 µg g^−1^ (with ranges extending to 0.106–0.164 µg g^−1^), and median and range for Y were 0.014 and 0.003–0.104 µg g^−1^ [49]. In addition, schoolchildren from Lovozero (Murmansk region, Russia; 7–15 years), located near REE-bearing ore extraction/processing in the Kola Peninsula [13], showed higher median REE concentrations than those observed in Alcalá (median [IQR], µg g^−1^): La 0.027 (0.020–0.036), Ce 0.030 (0.030–0.041), Pr 0.004 (0.004–0.005), Nd 0.015 (0.013–0.017), Gd 0.016 (0.010–0.028), Er 0.008 (0.007–0.009), and Y 0.016 (0.013–0.019). Likewise, studies in other regions have reported substantially higher summed REE burdens in children’s hair than those implied by our median REE profile (e.g., total REEs ~0.8–44 µg g^−1^ in a paediatric cohort from Madagascar), reinforcing that our data do not suggest elevated REE exposure in this population [53].

Conversely, substantially higher hair REE concentrations have been reported in clearly impacted settings. Tong et al. [54] quantified scalp-hair REEs in schoolchildren aged 11–15 years from two mining-area villages (M1, M2) and two local comparison villages (C1, C2) in southern China. Light REEs were markedly elevated in mining villages: geometric mean La was 0.89 and 0.45 µg g^−1^ in M1 and M2, respectively, versus 0.12 and 0.11 µg g^−1^ in C1 and C2; corresponding Nd geometric means were 0.67 and 0.28 µg g^−1^ (M1/M2) versus 0.12 and 0.09 µg g^−1^ (C1/C2). Comparable elevation patterns were reported for other REEs (e.g., Ce and Pr), reinforcing that the contrast with Alcalá reflects differences in overall environmental loading rather than an isolated element. Consistently, Tong et al. [54] also reported very wide ranges for some LREEs in the mining area (e.g., La 0.14–6.93 mg g^−1^ and Nd 0.09–5.27 mg g^−1^; i.e., 140–6930 and 90–5270 µg g^−1^, respectively), underscoring the magnitude contrast relative to Alcalá’s low-level profile.

A similar pattern emerges from hair REE measurements reported for residents of Bayan Obo (China; 2–93 years), the world’s largest REE mining region, compared with a non-exposed reference population from Hohhot. Median concentrations (µg g^−1^; Bayan Obo vs. Hohhot) were reported as: La 0.1431 vs. 0.0503; Ce 0.1606 vs. 0.0690; Pr 0.0256 vs. 0.0101; Nd 0.1302 vs. 0.0633; Eu 0.0040 vs. 0.0019; Gd 0.0126 vs. 0.0030; Er 0.0040 vs. 0.0013; and Y 0.0014 vs. 0.0004 [2]. Together, these external benchmarks indicate that the Alcalá dataset is more consistent with diffuse, low-intensity urban background inputs than with any high-loading context.

From an exposure-pathway standpoint, in a non-mining urban environment such as Alcalá de Henares, plausible contributors include resuspended road/soil dust, traffic-related particulates, indoor dust (including consumer products and electronics-associated sources), and diet [10]. In addition, “background” hair REE signals may be influenced by micro-sources and handling artefacts (e.g., Ce as a glass-polishing agent; sporadically elevated REEs in smokers linked to lighter flints), underscoring the need to interpret low-level hair REEs as a screening biomonitoring signal strengthened by coherent mixture structure rather than as a definitive proxy of internal dose for every individual element.

Shale normalisation (EUS/PAAS/WSH) was used here as a diagnostic of REE pattern shape rather than magnitude, and the median patterns were broadly subparallel across normalisers, indicating that qualitative fractionation features are not materially sensitive to the chosen reference composite (Figure S1). Consistent with this robustness, La and Ce anomalies showed similar central tendencies across EUS/PAAS/WSH (Figure S1; Tables S1 and S2). In children, median anomalies were La/La* ≈ 0.78–0.83 and Ce/Ce* ≈ 0.69–0.74 across normalisers, and adolescents showed comparable medians (La/La* ≈ 0.67–0.72; Ce/Ce* ≈ 0.67–0.73), indicating that La and Ce are, on average, not positively “enriched” relative to the smooth REE pattern predicted from Pr–Nd. Importantly, both anomalies exhibit wide ranges (upper-tail values up to ~25–41 in the strict-valid subset), which is expected when ratios are computed from very low shale-normalised abundances and can be driven by small denominators and residual heterogeneity near reporting limits; accordingly, interpretation should prioritise the distributional centre (median/IQR) and treat extreme anomalies as episodic/upper-tail behaviour rather than as evidence of systematic geochemical decoupling in this low-level urban baseline dataset.

### 4.2. Emerging and Technology-Related Elements in Hair: Children and Adolescents

In contrast to the internally coherent REE suite, the emerging/technology-related elements measured in this study showed highly heterogeneous detectability, which necessarily constrains inference. In both children and adolescents, Sb and U were quantified in most samples, but median concentrations were low (children: Sb 0.033 µg g^−1^, U 0.011 µg g^−1^; adolescents: Sb 0.0092 µg g^−1^, U 0.016 µg g^−1^), while Bi showed similarly low central tendency (children: 0.006 µg g^−1^; adolescents: 0.002 µg g^−1^). However, most analytes exhibited extensive left-censoring (Rh, Pd, Th, and Ir), while Pt was not detected in hair. These detection patterns are consistent with a scenario in which a small number of elements reflect relatively ubiquitous low-level exposure (or mixed endogenous–exogenous contributions), while others are present at or below reporting limits for most individuals, limiting the stability and interpretability of inter-element relationships.

For low-detectability elements, Rh, Pd, Ir, and Pt (and, in some studies, Th), published hair datasets in healthy children and adolescents are scarce and frequently dominated by left-censored results, meaning that cross-study comparisons are often not feasible or are statistically unstable even when these elements are reported.

In general, levels detected in Alcalá individuals were lower than those reported as reference values in hair in the Spanish young and general health population. Thus, Bi medians (children: 0.006 µg g^−1^; adolescents: 0.002 µg g^−1^) were lower than the reference range proposed in hair from children residing in the capital of Spain, Madrid city (<0.01–0.29 µg g^−1^), although interpretation should remain cautious because Bi distributions are sensitive to left-censoring and reporting limits [55]. For U, Spanish paediatric hair data are available mainly from specific environmentally pressured contexts; for example, children from the Flix area (Catalonia) showed median U concentrations of 0.048 µg g^−1^ in boys and 0.019 µg g^−1^ in girls [56]. In Alcalá, U medians were lower (children: 0.011 µg g^−1^; adolescents: 0.016 µg g^−1^), supporting an exposure profile consistent with diffuse, low-intensity urban background inputs rather than a dominant point source.

In a non-contaminated local area of southeast Spain (Elche/Alicante), Ruiz et al. [15] reported children’s hair reference values (P5–P95) of 0.001–0.02 µg g^−1^ for Bi and <0.001 µg g^−1^ for U. Bi medians observed in Alcalá fall within this Spanish reference interval, whereas U was quantified in essentially all children and at higher central values, which may reflect genuine spatial/temporal variability in background uranium exposure and/or (importantly) differences in analytical sensitivity, censoring structure, and study design that limit strict numeric comparability across cohorts. Complementarily, a Basque Country population study underscored the analytical constraints for Sb in hair at background levels, noting that Sb was analysed but remained below the LoD in a large fraction of samples (0.01 µg g^−1^, ~85%), highlighting that cross-study interpretation for low-level “emerging” analytes is highly dependent on LoDs and the proportion of left-censored observations [57].

These magnitudes are consistent with a low-intensity urban exposure profile when contextualised against European hair biomonitoring evidence. In Sicilian adolescents (11–14 years) living near petrochemical/industrial areas (Augusta, Gela, Pace del Mela), median hair Sb and U were generally higher than in Alcalá, with clear between-town variability [58]: for Sb, medians were 0.02 µg g^−1^ in the pooled industrial group and 0.01, 0.02, and 0.04 µg g^−1^ in each town, respectively; for U, medians were 0.03 µg g^−1^ for the group and 0.01, 0.03, and 0.05 µg g^−1^ across the same towns. In the corresponding suburban control group, median Sb and U were 0.04 and 0.02 µg g^−1^, respectively. By comparison, Alcalá adolescents show lower median Sb (0.0092 µg g^−1^) and lower median U (0.016 µg g^−1^), aligning more closely with a diffuse urban background profile than with settings influenced by major petrochemical/industrial emissions.

A similar pattern is apparent for Rb, which provides a useful additional European benchmark [58]: Sicilian adolescents living near petrochemical plants showed medians of 0.01 µg g^−1^ (whole group), 0.01 µg g^−1^ (Augusta and Gela), and 0.02 µg g^−1^ (Pace del Mela), concentrations that were similar to adolescents living in towns used as controls (suburban; 0.01 µg g^−1^). In Alcalá, adolescent Rb concentrations are lower (median 0.004 µg g^−1^), again consistent with low-intensity urban background inputs rather than a dominant industrial point source. Finally, broader European reference evidence supports this interpretation: an Italian reference-interval study in adolescents 11–13 years old from Palermo (Sicily) reported sex-stratified coverage intervals for both Rb (0.0002–0.06), Sb (0.0002–0.11), and U (0.0001–0.11; all in µg g^−1^) [59], placing Alcalá medians well within typical European non-occupational ranges.

Platinum was not detected in either cohort, and Rh/Ir were measurable only in a small minority of samples (Rh detected in 6 participants per cohort; Ir detected in only one child and in no adolescents), indicating that PGE loading was generally low and/or close to analytical reporting limits in this urban setting. Importantly, this pattern is consistent with non-occupational hair biomonitoring studies in which Pd and Pt typically occur at sub-ng g^−1^ to low-ng g^−1^ levels, whereas Rh and Ir are frequently below detection in general populations, particularly in youth. For example, in Sicilian adolescents (11–14 years old) living in urban (Palermo), control (Lentini), and petrochemical/industrial settings, median Pd concentrations ranged from 0.0006 to 0.00694 µg g^−1^ and median Pt from 0.00012 to 0.00156 µg g^−1^ [28]. In this context, the absence of Pt detections in Alcalá is analytically plausible because our cohort-specific Pt LoDs (0.005–0.0063 µg g^−1^) exceed the central values reported in comparable European adolescent cohorts. Notably, hair sampling occurred in 2001, during the expansion of catalyst-equipped petrol passenger car fleets following Euro 1 introduction (early 1990s), with Eurostat estimates indicating that by 2001 the share of petrol passenger cars fitted with catalytic converters was ~42% in Spain and ~68% in Italy (EU-15 ~72%) [60,61]. Given that traffic-related aerosols and resuspended road dust are the dominant diffuse urban pathways for PGEs, the Alcalá profile is most consistent with low-intensity, diffuse urban background inputs rather than a major local point source.

Similarly, a Swedish multi-element hair dataset reported median (range) concentrations in the general population (all in µg g^−1^) of Bi (0.009, 0.002–0.255), Ir (0.00001, <0.00001–0.000045), La (0.018, 0.0046–0.106), Pd (0.00006, <0.00006–0.0021), Pt (0.00008, 0.00002–0.00061), Rb (0.060, 0.012–0.482), Sb (0.017, 0.007–0.122), Th (0.0010, 0.0003–0.0044), and U (0.036, 0.006–0.436) [49]. These values provide a northern European benchmark and support the interpretation that Alcalá adolescents are not elevated relative to background distributions, particularly for U, Bi, Sb, and Rb. Together, these comparisons suggest that emerging-element exposure in Alcalá is more plausibly driven by diffuse urban sources (e.g., resuspended dust, traffic-related particulates, indoor dust/consumer-product contributions, and diet) than by any dominant point source.

Finally, the weak soil–hair concordance observed for the overlapping soil/hair analytes (Sb and Rh) indicates that topsoil gradients are unlikely to be the dominant determinant of individual hair burdens in either cohort. Where significant associations occurred, they were generally weak, sex-dependent, and not consistently element-homologous, reinforcing that alternative exposure pathways—such as inhalation of traffic-related aerosols, resuspended dust, indoor environments, diet, and consumer products—are likely to play a more prominent role than direct soil contact alone in shaping individual exposure profiles. This interpretation supports prioritising a multi-pathway urban exposure framework for the emerging elements in Alcalá, rather than attributing hair burdens to any single environmental compartment.

### 4.3. Age-Related Differences

Age-related differences in hair concentrations should be interpreted in the context of both behaviour/biology and the strong left-censoring affecting several analytes. In our cohort (children 6–9 vs. adolescents 13–16 years), REEs were quantified far more frequently in children, whereas adolescent REE data were dominated by non-detects; nevertheless, for several light REEs, concentrations were higher in adolescents when detectable (Ce, La, Pr, Nd; also Gd), while Er showed a contrasting pattern. A methodological contribution to these contrasts is plausible because adolescent subsamples required higher dilution (smaller available hair mass), which slightly increased instrumental LoDs despite identical ICP-MS conditions, mechanically reducing detection frequencies in the older group.

Published evidence indicates that age–REE relationships in hair are highly context-dependent and may differ between high-loading and background settings. In a rare-earth mining/smelting region, Dai et al. [2] reported decreasing Ce and Nd in children/young people with increasing age in the smelting area, but increasing Ce with age in adults, and an age-increase pattern already in younger ages in the reference area; they also highlighted that age and location were more influential than sex for hair REE burdens. To explain age-related decreases in high-exposure settings, these authors emphasised behavioural drivers, particularly declining hand-to-mouth contact and dust ingestion with increasing age, supported by a synthesis showing that indoor/outdoor hand-to-mouth frequencies fall across childhood [62]. They also noted that toxicokinetics may contribute, citing similar elimination patterns for Ce/Pr/Nd/Y in a biologically based toxicokinetic model [63] and broader evidence that REE properties may influence half-lives and accumulation patterns [49]. Consistent with this exposure-route logic, a paediatric cohort in Madagascar showed weak negative correlations between age and Ce (and also La and Nd) [53], and markedly elevated REEs have been reported in very young children (0–3 years) living closest to REE mining sources, with children exceeding their mothers [13,64].

Conversely, several studies in lower-exposure/background contexts—particularly across adulthood—report age-related increases for some REEs, suggesting that older groups may show higher concentrations when exposure is diffuse and low-level. In healthy adults (15–55 years), Zaichick and Zaichick [65] observed tendencies for Ce and La (and Th) to increase with age (more evident in females), while noting that some REEs were frequently below detection/only reported as upper limits, underscoring the role of analytical sensitivity and censoring. In a large sample of adult women, Skalnaya et al. [66] found only weak age–La associations in unadjusted analyses (and no clear independent age signal after multivariable modelling), illustrating that age trends may be subtle and confounded by covariates. At the extreme end of the lifespan, centenarian data suggest that age–REE patterns can reverse again; thus, Zhang et al. [67] reported decreasing trends with age for most REEs (except Ce), indicating that hair REE–age relationships are not expected to be linear across decades.

Taken together, the literature supports interpreting our child–adolescent pattern as reflecting the combined influence of (i) stronger dust/contact-mediated micro-exposure pathways at younger ages [2,62,64]; and (ii) heterogeneous time–activity patterns and upper-tail behaviour in a minority of individuals, which could yield higher concentrations among detectable values in adolescents for some light REEs [2,65]. In our low-level urban baseline dataset, this interpretation must also account for distributional/measurement effects, because slightly higher LoDs in adolescents due to dilution requirements can depress detection frequencies and amplify the influence of a small number of quantifiable values. Accordingly, age comparisons for REEs are best framed in terms of detectability and upper-tail behaviour rather than as a monotonic shift in central tendency.

For the technology/traffic-related suite, age contrasts should be interpreted primarily as distributional differences under heavy left-censoring, rather than as simple shifts in “typical” concentrations. In our cohort, children showed clearly higher Bi and Sb (Table 9), a pattern that is biologically and behaviourally plausible because younger children typically experience stronger contact-mediated exposure (closer interaction with indoor settled dust and higher non-dietary ingestion via hand-to-mouth activity). This interpretation is consistent with broader paediatric hair evidence indicating substantial variability across childhood and adolescence within single regions [55], and with age-stratified urban evidence reporting higher Pt and Sb in individuals aged <15 years than in older groups [68].

In contrast, Pd exhibited a modest but significant age effect expressed in the upper tail (higher P97.5 in adolescents than in children; Table 9), which is mechanistically plausible for a traffic/urban-activity tracer given established links between Pd/Pt and catalytic-converter emissions and re-suspension in road/urban dust [60,69]. Consistent with this, Lo Medico et al. [28] reported measurable Pd and Pt in scalp hair of adolescents (11–14 years) with site-dependent medians and wider reference intervals in anthropic settings, supporting the view that adolescent mobility and microenvironmental heterogeneity may manifest primarily as upper-tail behaviour for PGEs rather than as a uniform mean shift.

Rubidium, U, and Th did not show evidence of age-related differences, which is compatible with the expectation that, within a narrow paediatric window (6–16 years) and under strong censoring, age patterns may be weak or inconsistent across elements with different exposure routes. This contrasts with adult-focused evidence where age dependencies can be clearer; for example, an adult woman’s ultra-trace study reported age-associated increases in hair Rb and Sb [66]. Overall, the observed age-related patterns for emerging elements would most plausibly be interpreted as reflecting the combined influence of (a) comparatively greater contributions from contact- and dust-mediated microenvironments in younger children, potentially yielding higher hair Bi and Sb; and (b) more heterogeneous, activity- and mobility-related exposure profiles in adolescents, which could preferentially manifest as upper-tail increases for traffic-associated tracers such as Pd. This interpretation should, however, be regarded as provisional, given that sparse detectability for several PGEs and element-specific LoDs could materially shape both the apparent magnitude and the detectability structure of age contrasts.

### 4.4. Residential-Zone Contrasts

Zone-specific contrasts should be interpreted as differences in environmental microenvironments (soil/dust reservoirs and local emissions) in the presence of substantial left-censoring for several analytes; accordingly, Table 10 (children) and Table 11 (adolescents) summarise these spatial patterns using KM means and corresponding statistical comparisons, rather than relying solely on conventional measures of central tendency.

In children, statistically significant between-zone differences were observed for Ce, Er, Gd, and Pr (Table 10). Cerium was higher in the more urban/industrial areas (Zones II–IV) than in the greener low-density area (Zone I), whereas Er showed the opposite pattern, with the highest values in Zone I and markedly lower levels in Zones II–IV; Gd was also highest in Zone I, and Pr peaked in the industrial zone (Zone IV). This combination of (i) higher Ce in the denser/industrial zones and (ii) higher Er/Gd in the greener zone is compatible with a mixed source structure. First, Ce enrichment in more urbanised settings could plausibly reflect stronger contributions from traffic-related particulate reservoirs and re-suspension, and potentially Ce-bearing combustion-derived particles where cerium oxide is used in some diesel fuel-additive technologies [70,71,72]. Second, the inverse pattern for Er (and the higher Gd in Zone I) is consistent with a stronger geogenic “background” influence in greener/low-density areas where soil contact and local parent-material signals may dominate relative to anthropogenic inputs. This interpretation would also be coherent, as the Zone I topsoil geochemistry would have indicated comparatively higher REE-related tracers (e.g., Y) than the denser urban districts (see Figure 4/Table S3).

In contrast, emerging elements (including Sb and U) did not show statistically significant zonal effects in children (Table 10). In practice, the absence of significant zonal effects for some emerging elements in children may reflect that individual-level determinants (e.g., time–activity patterns and household-level indoor dust reservoirs) contribute substantially to exposure variability, while the strong left-censoring for several analytes may reduce sensitivity to detect modest spatial contrasts even when they exist [41,43,73,74].

In adolescents, the clearest zonal separation was observed for Rb, driven by markedly elevated values in Zone IV (industrial facilities) compared with Zones I–III (Table 11). Antimony also differed by zone, with the highest KM mean in the dense urban district (Zone II), while Th showed a significant overall zone effect with slightly higher KM means in Zones II/IV than in Zones I/III, similarly to the distribution observed for Sb. By contrast, U did not differ significantly across zones, although the numerically higher KM mean in the heavy-traffic corridor (Zone III) suggests that the zonal signal for U was concentrated in the upper tail (Table 11).

Benchmarking these patterns against the available hair literature supports the plausibility of (at least) two spatially structured factors in adolescents: an “urban/traffic” component and an “industrial” component. In Sicilian adolescents living near petrochemical/industrial areas, Varrica et al. [58] distinguished an urban factor that included Sb (among other elements) and an industrial factor that included U (among other elements), showing that adolescents’ hair profiles can be separated by residential context in ways consistent with local emissions and particulate reservoirs. Likewise, Lo Medico et al. [28] reported measurable Pd and Pt in adolescents’ scalp hair and differences between urban and industrial contexts, supporting the broader inference that local microenvironments can modulate hair burdens for technology/traffic-linked tracers. Taken together, these studies are directionally consistent with our observation that Sb peaks in the dense urban district (Zone II), while an industrial-area signal emerges strongly for at least one element (Rb) and is detectable for Th (Table 11).

Mechanistically, the adolescent peak of Sb in Zone II (dense urban; Table 11) is compatible with the well-documented enrichment of Sb in non-exhaust traffic emissions and its accumulation in road dust/indoor dust reservoirs, particularly via brake-wear–related inputs and subsequent re-suspension in compact street canyons [75,76]. Importantly, this interpretation is also consistent with the fact that the zone pattern in hair does not simply mirror topsoil: in our topsoil dataset, Sb showed a patchy structure with quantifiable values mainly in Zones I and III, whereas Zones II and IV were largely non-detectable at the applied LoD (Table S3).

Together, these observations could support the view that the Zone II Sb elevation in adolescents likely reflects a predominantly urban particulate microenvironment signal (resuspended road dust and indoor dust infiltration) rather than direct coupling to residential topsoil concentrations, and they reinforce the broader expectation that traffic-related tracers (including PGEs in road dust/air) can be spatially heterogeneous at neighbourhood scale [60,77].

Overall, the zone signal across our cohort is most coherently interpreted as the superposition of at least two components. First, a geogenic/background component appears more evident for some REEs in children, where Er and Gd were highest in the greener low-density area (Zone I) while Ce was higher in the more urban/industrial zones (Zones II/IV) (Table 10). Second, anthropogenic microenvironment components become clearer in adolescents for selected elements, including an urban Sb signal (highest in Zone II) and a strong industrial-area signal for Rb (marked elevation in Zone IV), with Th also showing a modest but significant enrichment in Zones II/IV relative to Zones I/III (Table 11). Because several analytes remain heavily left-censored, these contrasts should be framed explicitly as distributional differences that may be driven by (i) shifts in detectability above the LoD and/or (ii) changes in the upper tail attributable to a subset of more exposed individuals, rather than as uniform shifts across the entire concentration distribution.

A testable follow-up is therefore to model zone jointly with individual covariates (sex, time–activity proxies) and to evaluate whether zone modifies soil–hair associations for the key tracers highlighted by the zone analysis (e.g., Sb, Th, and REE indicators), using methods that explicitly accommodate left-censoring. 

## 5. Conclusions

This work delivers one of the earliest European paediatric scalp-hair datasets to jointly characterise rare earth elements (REEs) and technology-related trace metals in a non-mining urban environment. By analysing samples collected in 2001 from children and adolescents in Alcalá de Henares (Spain), the study establishes a robust “pre-diffusion” baseline that predates the widespread expansion of REE-enabled consumer technologies and provides a rare reference point for interpreting contemporary exposome signals.

Across the REE suite, children showed frequent quantification and a coherent within-suite correlation structure, consistent with shared sources and/or convergent exposure pathways. In adolescents, REE concentrations were predominantly near analytical reporting limits, indicating that apparent age differences are best interpreted through detectability and upper-tail behaviour rather than shifts in central tendency. Shale-normalised patterns were stable across alternative normalisation references, while La/Ce anomaly distributions were consistently centred below unity; the small set of extreme anomaly values is most parsimoniously explained by ratio instability at very low concentrations rather than systematic geochemical fractionation.

For technology-related elements, Sb and U emerged as the most consistently quantified in both cohorts, whereas platinum-group elements were largely below detection, underscoring the need for conservative inference focused on distributional descriptors and reference limits rather than hypothesis-driven comparisons for low-detectability analytes. Spatial contrasts by residential zone and weak concordance between hair and topsoil for overlapping tracers point to multiple microenvironmental contributors—particularly indoor dust and traffic-related aerosols in addition to soil contact—shaping inter-individual variability. The resulting percentile-based reference values (P95 with confidence intervals) are positioned as population reference limits (not health-based thresholds) and offer a defensible benchmark for future temporal comparisons as urban emission profiles and REE-related uses continue to evolve.

## Figures and Tables

**Table 1:** Concentrations of metals and metalloids (µg/g) in hair of **children** from Alcalá de Henares, Spain.

Element	N	% < LoD	LoD	A.M.	G.M.	Median	IQR	P95	CI-PP95	Range
**Bi**	69	42.5	0.005	0.035 ± 0.119	0.006	0.006	0.005, 0.016	0.094	0.023–0.172	0.005–0.996
**Ce**	95	20.8	0.0048	0.0141 ± 0.0138	0.0099	0.0109	0.0053, 0.0175	0.0310	0.0231–0.0414	0.0050–0.1185
**Er**	81	32.5	0.00025	0.00047 ± 0.00030	0.00034	0.00035	0.00025, 0.00059	0.0011	0.00069–0.00129	0.00026–0.00157
**Gd**	97	19.2	0.00039	0.00087 ± 0.00055	0.00069	0.00068	0.00045, 0.00102	0.00221	0.00137–0.00230	0.00038–0.00303
**Ir**	1	99.2	0.00181	/	0.00036	/	/	/	/	0.00231
**La**	77	35.8	0.0055	0.0106 ± 0.0111	0.0071	0.0072	0.0055, 0.0116	0.0222	0.0145–0.0277	0.0055–0.1043
**Nd**	86	28.3	0.00282	0.0055 ± 0.0033	0.0042	0.0042	0.0028, 0.0066	0.0124	0.0081–0.0126	0.0028–0.0201
**Pd**	12	90.0	0.00071	/	0.0002	/	/	0.00096	0.00111 *	0.00072–0.00492
**Pr**	99	17.5	0.00055	0.00168 ± 0.00120	0.00126	0.00137	0.00074, 0.00213	0.00399	0.00297–0.00457	0.00056–0.00777
**Pt**	0	100	0.00511	/	/	/	/	/	/	/
**Rb**	27	77.5	0.0124	0.0336 ± 0.0222	0.0300	0.0298	0.0225, 0.0373	0.0671	0.0458–0.0783	0.0105–0.2199
**Rh**	6	95.0	0.00176	/	0.00015	/	/	0.0018	0.0029 *	0.0018–0.00984
**Sb**	119	0.8	0.004	0.060 ± 0.081	0.036	0.033	0.017, 0.068	0.215	0.115–0.309	0.005–0.604
**Th**	5	95.8	0.00601	/	0.0001	/	/	/	0.0085 *	0.0076–0.0454
**U**	120	0	0.0008	0.021 ± 0.043	0.012	0.011	0.007, 0.020	0.052	0.037–0.077	0.001–0.367
**Y**	0	100	0.00560	/	/	/	/	/	/	/

N = number of samples above LoD; % < LoD = percentage below limit of detection; A.M. = arithmetic mean (results are presented as mean values ± S.D.); G.M. = geometric mean; IQR = interquartile range (P25, P75); P95 = 95th percentile; CI-PP95 = 95% confidence interval for 95PP (population percentile); * = 97.5th percentile; / = the data is not reporting enough information to determine this parameter.

**Table 2:** Concentrations of metals and metalloids (µg/g) in hair of **boys** from Alcalá de Henares, Spain.

Element	N	% < LoD	LoD	A.M.	G.M.	Median	IQR	P95	CI-PP95	Range
**Bi**	27	46.0	0.005	0.038 ± 0.149	0.005	0.006	0.005, 0.013	0.080	0.013–0.361	0.005–0.996
**Ce**	37	26.0	0.0048	0.0167 ± 0.0192	0.0100	0.0109	0.0048, 0.0190	0.0416	0.0198–0.0720	0.0054–0.1185
**Er**	32	36.0	0.00025	0.00049 ± 0.00037	0.00033	0.00031	0.00025, 0.00054	0.00143	0.00065–0.00147	0.00026–0.00157
**Gd**	37	26.0	0.00039	0.00083 ± 0.00058	0.00062	0.00058	0.00039, 0.00100	0.00207	0.00113–0.00294	0.00041–0.00303
**Ir**	0	100	0.00181	/	/	/	/	/	/	/
**La**	32	36.0	0.0055	0.01199 ± 0.01548	0.00712	0.00711	0.00553, 0.01184	0.03730	0.01336–0.04342	0.00555–0.10435
**Nd**	37	26.0	0.00282	0.00587 ± 0.00395	0.00438	0.00389	0.00282, 0.00714	0.01576	0.00805–0.01651	0.00306–0.02014
**Pd**	6	88.0	0.00071	/	0.00015	/	/	0.00110	0.00112 *	0.00091–0.00492
**Pr**	41	18.0	0.00055	0.0018 ± 0.0015	0.0013	0.0014	0.0008, 0.0022	0.0046	0.0026–0.0054	0.0006–0.0078
**Pt**	0	100	0.00511	/	/	/	/	/	/	/
**Rb**	6	88.0	0.0124	/	0.0051	/	/	0.0182	0.0191 *	0.0135–0.0300
**Rh**	4	92.0	0.00176	/	0.0005	/	/	0.0024	0.0033 *	0.0018–0.0037
**Sb**	50	0	0.004	0.070 ± 0.067	0.047	0.048	0.021, 0.090	0.223	0.127–0.301	0.010–0.301
**Th**	4	92.0	0.00601	/	0.0005	/	/	0.0081	0.0102 *	0.0076–0.0454
**U**	50	0	0.0008	0.012 ± 0.012	0.008	0.008	0.005, 0.014	0.034	0.020–0.054	0.001–0.065
**Y**	0	100	0.00560	/	/	/	/	/	/	/

N = number of samples above LoD; % < LoD = percentage below limit of detection; A.M. = arithmetic mean (results are presented as mean values ± S.D.); G.M. = geometric mean; IQR = interquartile range (P25, P75); P95 = 95th percentile; CI-PP95 = 95% confidence interval for 95PP (population percentile); * = 97.5th percentile; / = the data is not reporting enough information to determine this parameter.

**Table 3:** Concentrations of metals and metalloids (µg/g) in hair of **girls** from Alcalá de Henares, Spain.

Element	N	% < LoD	LoD	A.M.	G.M.	Median	IQR	P95	CI-PP95	Range
**Bi**	42	40.0	0.005	0.033 ± 0.094	0.007	0.006	0.005, 0.022	0.115	0.028–0.510	0.005–0.589
**Ce**	58	17.1	0.0048	0.0124 ± 0.0075	0.0097	0.0109	0.0055, 0.0168	0.0260	0.0207–0.0276	0.0050–0.0395
**Er**	49	30.0	0.00025	0.00045 ± 0.00023	0.00036	0.00035	0.00025, 0.00060	0.00104	0.00065–0.00113	0.00027–0.00117
**Gd**	60	14.3	0.00039	0.00090 ± 0.00053	0.00074	0.00073	0.00053, 0.00110	0.00227	0.00154–0.00230	0.00039–0.00230
**Ir**	1	98.6	0.00181	/	0.00046	/	/	/	/	0.00231
**La**	45	35.7	0.0055	0.0097 ± 0.0064	0.0072	0.0072	0.0055, 0.0114	0.0206	0.0127–0.0277	0.0057–0.0453
**Nd**	49	30.0	0.00282	0.0053 ± 0.0027	0.0041	0.0043	0.0028, 0.0065	0.0121	0.0070–0.0126	0.0028–0.0135
**Pd**	6	91.4	0.00071	/	0.00036	/	/	0.00076	0.00095 *	0.00072–0.00137
**Pr**	58	17.1	0.00055	0.0016 ± 0.0010	0.0012	0.0013	0.0007, 0.0021	0.0035	0.0024–0.0037	0.0006–0.0050
**Pt**	0	100	0.00511	/	/	/	/	/	/	/
**Rb**	21	70.0	0.0124	0.0107 ± 0.0072	0.0089	0.0089	0.0059, 0.0134	0.0242	0.0189–0.0311	0.0124–0.0594
**Rh**	2	97.1	0.00176	/	0.000018	/	/	/	0.00185 *	0.00208–0.00984
**Sb**	69	1.4	0.004	0.053 ± 0.089	0.030	0.027	0.016, 0.053	0.115	0.083–0.389	0.005–0.604
**Th**	1	98.6	0.00601	/	/	/	/	/	/	0.03249
**U**	70	0	0.0008	0.028 ± 0.054	0.016	0.014	0.009, 0.026	0.059	0.038–0.291	0.003–0.367
**Y**	0	100	0.00560	/	/	/	/	/	/	/

N = number of samples above LoD; % < LoD = percentage below limit of detection; A.M. = arithmetic mean (results are presented as mean values ± S.D.); G.M. = geometric mean; IQR = interquartile range (P25, P75); P95 = 95th percentile; CI-PP95 = 95% confidence interval for 95PP (population percentile); * = 97.5th percentile; / = the data is not reporting enough information to determine this parameter.

**Table 4:** Comparison of elemental concentrations (µg/g) in hair between **male and female children**.

Element	Boys	Girls	*p*-Value (Sex)
**Bi**	0.006 (0.005, 0.013)	0.006 (0.005, 0.022)	0.358
**Ce**	0.0109 (0.0048, 0.0190)	0.0109 (0.0055, 0.0168)	0.735
**Er**	0.00031 (0.00025, 0.00054)	0.00035 (0.00025, 0.00060)	0.615
**Gd**	0.00058 (0.00039, 0.00100)	0.00073 (0.00053, 0.00110)	0.179
**Ir**	ND	0.00231 †	/
**La**	0.00711 (0.00553, 0.01184)	0.0072 (0.0055, 0.0114)	0.970
**Nd**	0.00389 (0.00282, 0.00714)	0.0043 (0.0028, 0.0065)	0.692
**Pd**	P97.5: 0.00112	P97.5: 0.00095	0.466
**Pr**	0.0014 (0.0008, 0.0022)	0.0013 (0.0007, 0.0021)	0.570
**Rb**	P97.5: 0.0191	0.0089 (0.0059, 0.0134)	0.029 *
**Rh**	P97.5: 0.0029	P97.5: 0.00185	0.209
**Sb**	0.048 (0.021, 0.090)	0.027 (0.016, 0.053)	0.009 **
**Th**	P97.5: 0.0102	0.0325 †	0.358
**U**	0.008 (0.005, 0.014)	0.014 (0.009, 0.026)	0.0004 ***

Data reported as median values and the interquartile range (P25, P75); P97.5: 97.5th percentile; ND = not detected; † = only concentration detected; / = the data is not reporting enough information to determine this parameter; * *p* < 0.05; ** *p* < 0.01; *** *p* < 0.001.

**Table 5:** Concentrations of metals and metalloids (µg/g) in hair of **adolescents** from Alcalá de Henares, Spain.

Element	N	% < LoD	LoD	A.M.	G.M.	Median	IQR	P95	CI-PP95	Range
Bi	38	60.8	0.003	0.005 ± 0.011	0.002	0.002	0.001, 0.005	0.018	0.012–0.029	0.003–0.053
Ce	6	93.8	0.020	/	0.002	/	/	0.021	0.026 *	0.020–0.098
Er	8	91.8	0.0004	/	0.0001	/	/	0.0005	0.0008 *	0.0004–0.0009
Gd	1	98.97	0.0014	/	0.0001	/	/	/	/	0.00216
Ir	0	100	0.0025	/	/	/	/	/	/	/
La	8	91.8	0.0114	/	0.002	/	/	0.012	0.018 *	0.012–0.048
Nd	3	96.9	0.0097	/	0.0003	/	/	/	0.013 *	0.015–0.030
Pd	7	92.8	0.0012	/	0.0005	/	/	0.0015	0.0016 *	0.001–0.002
Pr	5	94.8	0.0026	/	0.0002	/	/	0.0027	0.0034 *	0.0028–0.0110
Pt	0	100	0.0063	/	/	/	/	/	/	/
Rb	25	74.2	0.011	0.017 ± 0.075	0.004	0.004	0.001, 0.012	0.066	0.036–0.122	0.011–0.106
Rh	6	93.8	0.00047	/	0.0001	/	/	0.0005	0.0006 *	0.0005–0.0013
Sb	96	1.03	0.002	0.013 ± 0.011	0.00998	0.0092	0.006, 0.016	0.039	0.026–0.047	0.003–0.080
Th	64	34.0	0.001	0.003 ± 0.004	0.002	0.002	0.001, 0.003	0.013	0.003–0.019	0.001–0.025
U	92	5.2	0.002	0.035 ± 0.094	0.013	0.016	0.005, 0.030	0.140	0.063–0.212	0.002–0.879

N = number of samples above LoD; % < LoD = percentage below limit of detection; A.M. = arithmetic mean (results are presented as mean values ± S.D.); G.M. = geometric mean; IQR = interquartile range (P25, P75); P95 = 95th percentile; CI-PP95 = 95% confidence interval for 95PP (population percentile); * = 97.5th percentile; / = the data is not reporting enough information to determine this parameter.

**Table 6:** Concentrations of metals and metalloids (µg/g) in hair of adolescent **male participants** from Alcalá de Henares, Spain.

Element	N	% < LoD	LoD	A.M.	G.M.	Median	IQR	P95	CI-PP95	Range
**Bi**	8	72.4	0.003	0.002 ± 0.003	0.001	0.001	0.0006, 0.003	0.0096	0.003–0.0099	0.003–0.012
**Ce**	4	86.2	0.020	/	0.006	/	/	0.023	0.047 *	0.020–0.098
**Er**	5	82.8	0.0004	/	0.0002	/	/	0.0007	0.00078 *	0.0004–0.0009
**Gd**	1	96.6	0.0014	/	0.0003	/	/	/	0.0016 *	0.0022
**Ir**	0	100	0.0025	/	/	/	/	/	/	/
**La**	4	86.2	0.0114	/	0.0034	/	/	0.0161	0.0276 *	0.0118–0.0482
**Nd**	2	93.1	0.0097	/	0.0009	/	/	0.0128	0.0194 *	0.0149–0.0301
**Pd**	2	93.1	0.0012	/	0.0005	/	/	0.0014	0.0015 *	0.0015–0.0016
**Pr**	3	89.7	0.0026	/	0.0005	/	/	0.0034	0.0058 *	0.0031–0.0110
**Pt**	0	100	0.0063	/	/	/	/	/	/	/
**Rb**	13	55.2	0.011	0.027 ± 0.030	0.010	0.013	0.006, 0.048	0.086	0.012–0.102	0.011–0.106
**Rh**	6	79.3	0.00047	0.00031 ± 0.00028	0.0003	0.0002	0.0001, 0.0004	0.0008	0.0005–0.0008	0.0005–0.0013
**Sb**	29	0	0.002	0.012 ± 0.014	0.009	0.008	0.006, 0.012	0.024	0.014–0.080	0.003–0.080
**Th**	19	34.5	0.001	0.0027 ± 0.0030	0.0016	0.0015	0.001, 0.003	0.008	0.003–0.008	0.001–0.016
**U**	25	13.8	0.002	0.018 ± 0.037	0.007	0.007	0.003, 0.016	0.066	0.017–0.066	0.002–0.197

N = number of samples above LoD; % < LoD = percentage below limit of detection; A.M. = arithmetic mean (results are presented as mean values ± S.D.); G.M. = geometric mean; IQR = interquartile range (P25, P75); P95 = 95th percentile; CI-PP95 = 95% confidence interval for 95PP (population percentile); * = 97.5th percentile; / = the data is not reporting enough information to determine this parameter.

**Table 7:** Concentrations of metals and metalloids (µg/g) in hair of adolescent **female participants** from Alcalá de Henares, Spain.

Element	N	% < LoD	LoD	A.M.	G.M.	Median	IQR	P95	CI-PP95	Range
**Bi**	30	55.9	0.003	0.006 ± 0.014	0.002	0.002	0.001, 0.006	0.022	0.013–0.039	0.003–0.053
**Ce**	2	97.1	0.020	/	0.0004	/	/	/	0.022 *	0.026–0.080
**Er**	3	95.6	0.0004	/	0.00006	/	/	/	0.00057 *	0.0005–0.0009
**Gd**	0	100	0.0014	/	/	/	/	/	/	/
**Ir**	0	100	0.0025	/	/	/	/	/	/	/
**La**	4	94.1	0.0114	/	0.0019	/	/	0.0115	0.0146 *	0.0115–0.0424
**Nd**	1	98.5	0.0097	/	0.00013	/	/	/	/	0.0208
**Pd**	5	92.6	0.0012	/	0.0005	/	/	0.0014	0.0016 *	0.0012–0.0020
**Pr**	2	97.1	0.0026	/	0.0001	/	/	/	0.0027 *	0.0028–0.0083
**Pt**	0	100	0.0063	/	/	/	/	/	/	/
**Rb**	12	82.4	0.011	/	0.0026	/	/	0.0411	0.0455 *	0.0128–0.0726
**Rh**	0	100	0.00047	/	/	/	/	/	/	/
**Sb**	67	1.5	0.002	0.013 ± 0.010	0.011	0.010	0.006, 0.016	0.039	0.024–0.041	0.003–0.047
**Th**	45	33.8	0.001	0.003 ± 0.005	0.001	0.002	0.001, 0.003	0.013	0.003–0.022	0.001–0.025
**U**	67	1.5	0.002	0.042 ± 0.109	0.017	0.020	0.006, 0.031	0.140	0.063–0.212	0.003–0.879

N = number of samples above LoD; % < LoD = percentage below limit of detection; A.M. = arithmetic mean (results are presented as mean values ± S.D.); G.M. = geometric mean; IQR = interquartile range (P25, P75); P95 = 95th percentile; CI-PP95 = 95% confidence interval for 95PP (population percentile); * = 97.5th percentile; / = the data is not reporting enough information to determine this parameter.

**Table 8:** Comparison of elemental concentrations in hair between **adolescent males and females** (median and IQR).

Element	Male	Female	*p*-Value (Sex)
**Bi**	0.001 (0.0006, 0.003)	0.002 (0.001, 0.006)	0.077
**Ca**	168.062 (140.468, 615.565)	366.964 (242.869, 652.407)	0.022 *
**Ce**	P97.5: 0.047	P97.5: 0.022	0.046 *
**Er**	P97.5: 0.00078	P97.5: 0.00057	0.039 *
**La**	P97.5: 0.0276	P97.5: 0.0146	0.188
**Nd**	P97.5: 0.0194	0.0208 †	/
**Pd**	P97.5: 0.0015	P97.5: 0.0016	0.937
**Pr**	P97.5: 0.0058	P97.5: 0.0027	0.125
**Rb**	0.013 (0.006, 0.048)	P97.5: 0.0455	0.001 **
**Sb**	0.008 (0.006, 0.012)	0.010 (0.006, 0.016)	0.190
**Th**	0.0015 (0.001, 0.003)	0.002 (0.001, 0.003)	0.770
**U**	0.007 (0.003, 0.016)	0.020 (0.006, 0.031)	0.005 **

Data reported as median values and the interquartile range (P25, P75); P97.5: 97.5th percentile; ND = not detected; † = only concentration detected; / = the data is not reporting enough information to determine this parameter; * *p* < 0.05; ** *p* < 0.01.

**Table 9:** Comparison of elemental concentrations (µg/g) in hair between **children and adolescents**.

Element	Children	Adolescents	*p*-Value (Age)
**Bi**	0.006 (0.005, 0.016)	0.002 (0.001, 0.005)	0.0000018 ***
**Ce**	0.0109 (0.0053, 0.0175)	P97.5: 0.026	0.0184 *
**Er**	0.00035 (0.00025, 0.00059)	P97.5: 0.0008	0.000000841 ***
**Gd**	0.00068 (0.00045, 0.00102)	0.00216†	0.0374 *
**Ir**	0.00231 †	/	/
**La**	0.0072 (0.0055, 0.0116)	P97.5: 0.018	0.00052 ***
**Nd**	0.0042 (0.0028, 0.0066)	P97.5: 0.013	0.0374 *
**Pd**	P97.5: 0.00111	P97.5: 0.0016	0.0424 *
**Pr**	0.00137 (0.00074, 0.00213)	P97.5: 0.0034	0.0215 *
**Pt**	/	/	/
**Rb**	0.0298 (0.0225, 0.0373)	0.004 (0.001, 0.012)	0.303
**Rh**	0.030 (0.022, 0.037)	P97.5: 0.00006	0.026 *
**Sb**	0.033 (0.017, 0.068)	0.0092 (0.006, 0.016)	2.14 × 10^−22^ ***
**Th**	P97.5: 0.0085	0.002 (0.001, 0.003)	0.498
**U**	0.011 (0.007, 0.020)	0.016 (0.005, 0.030)	0.549

Data reported as median values and the interquartile range (P25, P75); P97.5: 97.5th percentile; ND = not detected; † = only concentration detected; / = the data is not reporting enough information to determine this parameter; * *p* < 0.05; *** *p* < 0.001.

**Table 10:** Spatial (zone) differences in hair element concentrations in **children** (Alcalá de Henares).

Element	Zone	N	KM Mean	LCLMean	*p*-Value
**Bi**	I	24	0.019 ± 0.026	0.008	0.073
	II	60	0.056 ± 0.163	0.014	
	IV	36	0.010 ± 0.006	0.008	
**Ce**	I	24	0.008 ± 0.004	0.007 **a**	0.005 **
	II	60	0.015 ± 0.017	0.011 **b**	
	IV	36	0.016 ± 0.009	0.013 **b**	
**Er**	I	24	0.00073 ± 0.00031	0.00059 **a**	1.04 × 10^−10^ ***
	II	60	0.00034 ± 0.00022	0.00033 **b**	
	IV	36	0.00038 ± 0.00029	0.00034 **b**	
**Gd**	I	24	0.0012 ± 0.0007	0.0009 **a**	0.004 **
	II	60	0.0007 ± 0.0004	0.0006 **b**	
	IV	36	0.0009 ± 0.0005	0.0007 **ab**	
**La**	I	24	0.007 ± 0.003	0.006	0.086
	II	60	0.012 ± 0.015	0.008	
	IV	36	0.010 ± 0.006	0.008	
**Li**	I	24	0.014 ± 0.004	0.012	0.075
	II	60	0.010 ± 0.001	0.009	
	IV	36	0.016 ± 0.021	0.008	
**Nd**	I	24	0.0052 ± 0.0037	0.0036	0.667
	II	60	0.0055 ± 0.0030	0.0047	
	IV	36	0.0058 ± 0.0034	0.0046	
**Pd**	I	24	0.0007 ± 0.00004	0.00070	0.913
	II	60	0.0008 ± 0.00010	0.00077	
	IV	36	0.0009 ± 0.00069	0.00061	
**Pr**	I	24	0.0013 ± 0.0009	0.0009 **a**	0.012 *
	II	60	0.0016 ± 0.0012	0.0013 **a**	
	IV	36	0.0020 ± 0.0012	0.0016 **b**	
**Rb**	I	24	0.0136 ± 0.0012	0.013	0.465
	II	60	0.0142 ± 0.0040	0.013	
	IV	36	0.0152 ± 0.0086	0.012	
**Sb**	I	24	0.047 ± 0.055	0.024	0.513
	II	60	0.059 ± 0.072	0.040	
	IV	36	0.071 ± 0.106	0.035	
**U**	I	24	0.012 ± 0.008	0.009	0.129
	II	60	0.020 ± 0.039	0.010	
	IV	36	0.028 ± 0.060	0.008	

Zones correspond to the residential-area classification described in Methods and Figure 1. N = number of participants in each zone. For variables with left-censored observations (<LoD), zone-level central tendency was estimated using the Kaplan–Meier mean (KM Mean) (reported as mean ± dispersion as shown in the table); LCL Mean = lower 95% confidence limit of the KM mean. *p*-value denotes the overall comparison among zones using the Peto–Peto (modified Gehan–Wilcoxon) test for censored variables. Where the overall test was significant, post hoc pairwise comparisons were performed; different superscript letters (a, b, ab) indicate statistically significant differences between zones (same letter = not significantly different). *p*-values from multiple comparisons were adjusted using the Benjamini–Hochberg false discovery rate (FDR) procedure. Asterisks denote significance levels: * FDR-adjusted* *p* < 0.05; ** *p* < 0.01; *** *p* < 0.001.

**Table 11:** Spatial (zone) differences in hair element concentrations in **adolescents** (Alcalá de Henares).

Element	Zone	N	KM Mean	LCL Mean	*p*-Value
**Bi**	I	36	0.005 ± 0.005	0.003	0.490
	II	20	0.008 ± 0.003	0.006	
	III	24	0.007 ± 0.011	0.002	
	IV	17	0.005 ± 0.003	0.003	
**Rb**	I	36	0.013 ± 0.002	0.012 **a**	3.64 × 10^−22^ ***
	II	20	0.016 ± 0.018	0.003 **a**	
	III	24	0.015 ± 0.007	0.012 **a**	
	IV	17	0.050 ± 0.021	0.039 **b**	
**Sb**	I	36	0.011 ± 0.007	0.008 **a**	0.0096 *
	II	20	0.019 ± 0.017	0.011 **b**	
	III	24	0.010 ± 0.008	0.007 **a**	
	IV	17	0.013 ± 0.011	0.007 **ab**	
**Th**	I	36	0.0023 ± 0.0034	0.0011 **a**	0.0056 **
	II	20	0.0037 ± 0.0063	0.0062 **b**	
	III	24	0.0022 ± 0.0024	0.0011 **a**	
	IV	17	0.0037 ± 0.0035	0.0019 **b**	
**U**	I	36	0.025 ± 0.030	0.014	0.138
	II	20	0.015 ± 0.015	0.008	
	III	24	0.080 ± 0.176	0.004	
	IV	17	0.016 ± 0.009	0.012	

Zones correspond to the residential-area classification described in Methods and Figure 1. N = number of participants in each zone. For variables with left-censored observations (<LoD), zone-level central tendency was estimated using the Kaplan–Meier mean (KM Mean) (reported as mean ± dispersion as shown in the table); LCL Mean = lower 95% confidence limit of the KM mean. *p*-value denotes the overall comparison among zones using the Peto–Peto (modified Gehan–Wilcoxon) test for censored variables. Where the overall test was significant, post hoc pairwise comparisons were performed; different superscript letters (a, b, ab) indicate statistically significant differences between zones (same letter = not significantly different). *p*-values from multiple comparisons were adjusted using the Benjamini–Hochberg false discovery rate (FDR) procedure. Asterisks denote significance levels: * FDR-adjusted* *p* < 0.05; ** *p* < 0.01; *** *p* < 0.001.

## Data Availability

The data presented in this study are openly available in Open Science Framework (OSF) at https://osf.io/69v78/overview?view_only=f05b8ba753324acc9ae8f293231d37a8 (accessed on 4 February 2026).

## Supplementary Materials

The following supporting information can be downloaded at: https://www.mdpi.com/article/10.3390/jox16010038/s1, Supplementary data associated with this article include Tables S1–S3 and Figures S1 and S2, providing shale-normalised REE patterns, La/Ce anomaly distributions, and supporting soil/zone summaries. Figure S1. Shale-normalised rare earth element (REE) patterns in scalp hair by age group and shale reference composite (Alcalá de Henares, Spain). Figure S2. Lanthanum and cerium anomaly ratios (La/La* and Ce/Ce*) in scalp hair by age group and shale normaliser (Alcalá de Henares, Spain). Table S1. Shale-normalised REE values and La/Ce anomalies in hair of children from Alcalá de Henares (Spain), using EUS [38], PAAS [39] and WSH [38] normalisers. Table S2. Shale-normalised REE values and La/Ce anomalies in hair of adolescents from Alcalá de Henares (Spain), using EUS [38], PAAS [39] and WSH [38] normalisers. Table S3. Concentration of a number of elements in Alcalá soil samples according to urban zones (mg kg^−1^).
