# Supplementary material for: Rare Earth Elements and Technology-Related Trace Metals in Paediatric Scalp Hair: A 2001 Urban Baseline from Spain
Source: J Xenobiot. 2026 Feb 23;16(1):38. doi: 10.3390/jox16010038 (PMC12942144; doi:10.3390/jox16010038)
Supplement: Supplementary file 1 [file jox-16-00038-s001.zip › Supplementary tables REEs emerging elements manuscript_APF.docx]

## Table S1. Shale-normalised REE values and La/Ce anomalies in hair of children from Alcalá de Henares (Spain), using EUS [38], PAAS [39] and WSH [38] normalisers.

| **Element**  **(µg g^-1^)** | **EUS** | **PAAS** | **WSH** | **EUS** | | | **PAAS** | | | **WSH** | | |
| --- | --- | --- | --- | --- | --- | --- | --- | --- | --- | --- | --- | --- |
|  |  |  |  | **Median** | **IQR** | **Range** | **Median** | **IQR** | **Range** | **Median** | **IQR** | **Range** |
| **La** | 44.3 | 44.56 | 40.3 | 0.00017 | 0.00010, 0.00027 | 0.0000104-0.0023555 | 0.00017 | 0.00009, 0.00027 | 0.0000104-0.0023417 | 0.00019 | 0.00008, 0.00030 | 0.0000115-0.0025893 |
| **Ce** | 88.5 | 88.25 | 83.3 | 0.00013 | 0.00007, 0.00021 | 0.0000021-0.0013393 | 0.00013 | 0.00007, 0.00021 | 0.0000021-0.0013431 | 0.00013 | 0.00008, 0.00022 | 0.0000022-0.0014230 |
| **Pr** | 10.6 | 10.15 | 9.54 | 0.00014 | 0.00008, 0.00021 | 0.0000075-0.0007326 | 0.00015 | 0.00009, 0.00022 | 0.0000078-0.0007651 | 0.00016 | 0.00008, 0.00023 | 0.0000083-0.0008140 |
| **Nd** | 39.5 | 37.32 | 36.6 | 0.00012 | 0.00007, 0.00017 | 0.0000101-0.0005098 | 0.00012 | 0.00008, 0.00018 | 0.0000107-0.0005396 | 0.00013 | 0.00008, 0.00019 | 0.0000109-0.0005502 |
| **Gd** | 6.34 | 6.043 | 5.86 | 0.00011 | 0.00007, 0.00017 | 0.0000075-0.0004786 | 0.00011 | 0.00008, 0.00018 | 0.0000078-0.0005021 | 0.00012 | 0.00008, 0.00018 | 0.0000081-0.0005178 |
| **Er** | 3.43 | 3.075 | 3.3 | 0.00010 | 0.00006, 0.00018 | 0.0000108-0.0004569 | 0.00012 | 0.00007, 0.00020 | 0.0000121-0.0005096 | 0.00011 | 0.00008, 0.00018 | 0.0000112-0.0004748 |
| **Y** | 31.9 | 27.31 | 28.7 | / | / | / | / | / | / | / | / | / |
| **La *_N_*_,ref_ / La* *_N_*_,ref_** | | | | 0.8347 | 0.4324, 1.7852 | 0.0274-25.7559 | 0.8162 | 0.4228, 1.7456 | 0.0268-25.1841 | 0.7791 | 0.4036, 1.6663 | 0.0255-24.0400 |
| **Ce *_N_*_,ref_ / Ce* *_N_*_,ref_** | | | | 0.7433 | 0.5476, 1.0919 | 0.0994-9.5335 | 0.7234 | 0.5329, 1.0626 | 0.0968-9.2780 | 0.6903 | 0.5086, 1.0141 | 0.0924-8.8542 |

Values are dimensionless shale-normalised abundances (REE*_N_*) and dimensionless anomalies. Data are summarised as Median and the interquartile interval (P25–P75), with the full range (Min–Max). La and Ce anomalies were calculated as La/La* and Ce/Ce*, where expected values were derived from neighbouring shale-normalised REEs (La* = Pr*_N_*³/Nd*_N_*²; Ce* = Pr*_N_*²/Nd*_N_*; [37]). Anomalies were summarised using the same strict-valid subset used for plotting (rows failing validity criteria are treated as missing). “/” indicates not available (insufficient valid data).

## Table S2. Shale-normalised REE values and La/Ce anomalies in hair of adolescents from Alcalá de Henares (Spain), using EUS [38], PAAS [39] and WSH [38] normalisers..

| **Element**  **(µg g^-1^)** | **EUS** | **PAAS** | **WSH** | **EUS** | | | **PAAS** | | | **WSH** | | |
| --- | --- | --- | --- | --- | --- | --- | --- | --- | --- | --- | --- | --- |
|  |  |  |  | **Median** | **IQR** | **Range** | **Median** | **IQR** | **Range** | **Median** | **IQR** | **Range** |
| **La** | 44.3 | 44.56 | 40.3 | 0.000083 | 0.000045, 0.000143 | 0.0000003-0.0010872 | 0.000083 | 0.000045, 0.000143 | 0.0000003-0.0010809 | 0.000092 | 0.000031, 0.000158 | 0.0000004-0.0011952 |
| **Ce** | 88.5 | 88.25 | 83.3 | 0.000057 | 0.000035, 0.000133 | 0.0000039-0.0011052 | 0.000058 | 0.000035, 0.000133 | 0.0000039-0.0011084 | 0.000061 | 0.000031, 0.000141 | 0.0000042-0.0011742 |
| **Pr** | 10.6 | 10.15 | 9.54 | 0.000057 | 0.000024, 0.000098 | 0.0000009-0.0010396 | 0.000059 | 0.000025, 0.000103 | 0.0000010-0.0010857 | 0.000063 | 0.000030, 0.000109 | 0.0000010-0.0011551 |
| **Nd** | 39.5 | 37.32 | 36.6 | 0.000049 | 0.000022, 0.000119 | 0.0000002-0.0007622 | 0.000052 | 0.000023, 0.000126 | 0.0000002-0.0008067 | 0.000053 | 0.000030, 0.000129 | 0.0000002-0.0008226 |
| **Gd** | 6.34 | 6.043 | 5.86 | 0.000044 | 0.000025, 0.000078 | 0.0000040-0.0003412 | 0.000046 | 0.000027, 0.000082 | 0.0000042-0.0003580 | 0.000048 | 0.000030, 0.000085 | 0.0000043-0.0003692 |
| **Er** | 3.43 | 3.075 | 3.3 | 0.000039 | 0.000013, 0.000068 | 0.0000009-0.0002533 | 0.000043 | 0.000014, 0.000076 | 0.0000010-0.0002825 | 0.000040 | 0.000030, 0.000071 | 0.0000010-0.0002633 |
| **La *_N_*_,ref_ / La* *_N_*_,ref_** | | | | 0.72163 | 0.31411, 1.49778 | 0.00064-41.15133 | 0.70561 | 0.30714, 1.46453 | 0.00063-40.23776 | 0.67356 | 0.29318, 1.39799 | 0.00060-38.40980 |
| **Ce *_N_*_,ref_ / Ce* *_N_*_,ref_** | | | | 0.72541 | 0.50370, 1.03611 | 0.00588-25.08631 | 0.70597 | 0.49020, 1.00835 | 0.00572-24.41412 | 0.67372 | 0.46781, 0.96229 | 0.00546-23.29893 |

Values are dimensionless shale-normalised abundances (REE*_N_*) and dimensionless anomalies. Data are summarised as Median and the interquartile interval (P25–P75), with the full range (Min–Max). La and Ce anomalies were calculated as La/La* and Ce/Ce*, where expected values were derived from neighbouring shale-normalised REEs (La* = Pr*_N_*³/Nd*_N_*²; Ce* = Pr*_N_*²/Nd*_N_*; [37]). Anomalies were summarised using the same strict-valid subset used for plotting (rows failing validity criteria are treated as missing).

## Table S3. Concentration of a number of elements in Alcalá soil samples according to urban zones (mg kg^-1^).

| **Element** | **Zone 1** | **Zone 2** | **Zone 3** | **Zone 4** | ***P*** |
| --- | --- | --- | --- | --- | --- |
| **Pt** | 0.430 (<0.345-0.579) ^a^ | 0.795 (<0.345-1.448) ^a^ | 0.348 (0.246-0.491) ^a^ | 0.267 (0.111-0.640) ^a^ | 0.032 |
| **Rh** | 0.172 (0.101-0.294) | 0.264 ** | 0.185 (0.073-0.468) | 0.253 ** | / |
| **Sb** | 0.415 * | ND | 0.117 (0.063-0.218) | ND | / |
| **Y** | 7.111 (5.071-8.770) ^a^ | 4.638 (3.558-5.044) ^b^ | 4.801 (3.951-5.497) ^b^ | 5.514 (4.118-6.459) ^b^ | <0.0001 |

Results are presented as medians and IQR (all in mg kg^-1^); ND= Not detected. Different letters in the same row indicate significantly different values (*p*-value < 0.05). * High sample percentil 95; ** high sample percentil 97.5.

References

37. Barrat, J. A., & Bayon, G. (2024). Practical guidelines for representing and interpreting rare earth abundances in environmental and biological studies. *Chemosphere*, *352*, 141487. <https://doi.org/10.1016/j.chemosphere.2024.141487>

38. Bau, M., Schmidt, K., Pack, A., Bendel, V., & Kraemer, D. (2018). The European Shale: An improved data set for normalisation of rare earth element and yttrium concentrations in environmental and biological samples from Europe. *Applied Geochemistry*, *90*, 142-149. <https://doi.org/10.1016/j.apgeochem.2018.01.008>

39. Pourmand, A., Dauphas, N., & Ireland, T. J. (2012). A novel extraction chromatography and MC-ICP-MS technique for rapid analysis of REE, Sc and Y: Revising CI-chondrite and Post-Archean Australian Shale (PAAS) abundances. *Chemical Geology*, *291*, 38-54. <https://doi.org/10.1016/j.chemgeo.2011.08.011>
