# Supplementary material for: Rare Earth Elements and Technology-Related Trace Metals in Paediatric Scalp Hair: A 2001 Urban Baseline from Spain
Source: J Xenobiot. 2026 Feb 23;16(1):38. doi: 10.3390/jox16010038 (PMC12942144; doi:10.3390/jox16010038)
Supplement: Supplementary file 1 [file jox-16-00038-s001.zip › Figure_S1 REEs normalizados.docx]

**Figure S1.** Shale-normalised rare earth element (REE) patterns in scalp hair by age group and shale reference composite (Alcalá de Henares, Spain).

|  |  |
| --- | --- |
| **A** |  |
| **** | **** |
| **B** | **C** |

Median shale-normalised abundances (REE*_N_*) are shown for children (6–9 years; panel A; panel B reproduces the children’s curve on the same scale to facilitate visual comparison with adolescents) and adolescents (13–16 years; panel C). Normalisation was performed using three shale reference composites: European Shale (EUS; [38]), Post-Archean Australian Shale (PAAS; [39]) and World Shale (WSH; [38]). Element order follows increasing atomic number; values are plotted on a log y-axis to emphasise pattern shape (relative fractionation) rather than absolute magnitude. REE_N values were computed only for samples in which the corresponding element concentration was quantified (*i.e.*, values <LoD were not substituted), so the plotted medians reflect the quantifiable subset for each element and age group. Subparallel pattern shapes across normalisers indicate that qualitative REE fractionation is robust to the choice of reference composite in this low-level urban baseline dataset.

References

38. Bau, M., Schmidt, K., Pack, A., Bendel, V., & Kraemer, D. (2018). The European Shale: An improved data set for normalisation of rare earth element and yttrium concentrations in environmental and biological samples from Europe. *Applied Geochemistry*, *90*, 142-149. <https://doi.org/10.1016/j.apgeochem.2018.01.008>

39. Pourmand, A., Dauphas, N., & Ireland, T. J. (2012). A novel extraction chromatography and MC-ICP-MS technique for rapid analysis of REE, Sc and Y: Revising CI-chondrite and Post-Archean Australian Shale (PAAS) abundances. *Chemical Geology*, *291*, 38-54. <https://doi.org/10.1016/j.chemgeo.2011.08.011>
