# Supplementary material for: Rare Earth Elements and Technology-Related Trace Metals in Paediatric Scalp Hair: A 2001 Urban Baseline from Spain
Source: J Xenobiot. 2026 Feb 23;16(1):38. doi: 10.3390/jox16010038 (PMC12942144; doi:10.3390/jox16010038)
Supplement: Supplementary file 1 [file jox-16-00038-s001.zip › Figure_S2 La Ce anomalies.docx]

**Figure S2.** Lanthanum and cerium anomaly ratios (La/La* and Ce/Ce*) in scalp hair by age group and shale normaliser (Alcalá de Henares, Spain).

Box-and-whisker plots summarise distributions of La/La* and Ce/Ce* for (A) children (6–9 years) and (B) adolescents (13–16 years), calculated from shale-normalised REE abundances using EUS [38], PAAS [39] and WSH [38]. Expected values were estimated from neighbouring elements using the Pr–Nd geometric formulation (semi-log approach): La* *_N_*_,ref_ = (Pr *_N_*_,ref_)³ / (Nd *_N_*_,ref_)² and Ce* *_N_*_,ref_ = (Pr *_N_*_,ref_ ²) / Nd *_N_*_,ref_. Anomalies were computed under a strict-valid rule (only when La, Ce, Pr and Nd were all quantified in the same sample) to avoid mathematically unstable ratios driven by censoring at very low concentrations. Boxes show the interquartile range (P25–P75) with median line; whiskers extend to 1.5×IQR and points beyond are plotted as outliers. Values centred below unity indicate that, on average, La and Ce are not enriched relative to a smooth Pr–Nd-defined REE pattern, consistent with low-intensity background exposure conditions in this cohort.

References

38. Bau, M., Schmidt, K., Pack, A., Bendel, V., & Kraemer, D. (2018). The European Shale: An improved data set for normalisation of rare earth element and yttrium concentrations in environmental and biological samples from Europe. *Applied Geochemistry*, *90*, 142-149. <https://doi.org/10.1016/j.apgeochem.2018.01.008>

39. Pourmand, A., Dauphas, N., & Ireland, T. J. (2012). A novel extraction chromatography and MC-ICP-MS technique for rapid analysis of REE, Sc and Y: Revising CI-chondrite and Post-Archean Australian Shale (PAAS) abundances. *Chemical Geology*, *291*, 38-54. <https://doi.org/10.1016/j.chemgeo.2011.08.011>
